# Mo Dopant-Mediated Oxygen Vacancy Engineering for Enhanced Cathodic Activity in Protonic Ceramic Fuel Cells

**DOI:** 10.1021/acsami.6c06472

**Published:** 2026-07-07

**Authors:** Wenkai Yang, Yuting Li, Timileyin Aworinde, Shaikh Abdullah, Lakshya Mathur, Daofan Wang, Yue Bao, Linfeng Yu, Lourdes Vega, Chuancheng Duan, Sivaprakash Sengodan

**Affiliations:** † Department of Mechanical and Nuclear Engineering, 105955 Khalifa University, Abu Dhabi 127788, UAE; ‡ Department of Chemical and Petroleum Engineering, Khalifa University, Abu Dhabi 127788, UAE; § Research and Innovation Center on CO_2_ and Hydrogen (RICH), Khalifa University, Abu Dhabi 127788, UAE; ∥ Department of Chemical Engineering, 646883 University of Utah, Salt Lake City, Utah 84112, United States

**Keywords:** protonic ceramic fuel cells, triple conducting oxides, perovskite, ORR, cathodes, DFT

## Abstract

Protonic ceramic fuel cells (PCFCs) have attracted more interest than solid oxide fuel cells (SOFCs) due to their potential to be operated at lower temperatures, therefore, addressing the limitation of high-temperature oxygen ion conducting SOFC. Layered double perovskite with triple conducting (H^+^/O^2–^/e^–^) properties, such as PrBa_0.5_Sr_0.5_Co_1.5_Fe_0.5_O_5+δ_ (PBSCF), have demonstrated outstanding performance as cathodes in PCFCs. Our investigation on molybdenum-doped (Mo-doped) PBSCF showed that high valence Mo doping is an effective strategy for substantially promoting the formation of oxygen vacancy and accelerating the overall cathodic reaction. The improved oxygen reduction reaction (ORR)-related kinetics are reflected by the reduced polarization resistance (*R*_p_) of PBSCFM01 (0.052 Ω·cm^2^ at 650 °C) and the enhanced peak power density (PPD) of the PBSCFM01 cell (817 mW·cm^–2^ at 650 °C), corresponding to a 75% decrease in *R*_p_ and a 44% increase in PPD compared with pure PBSCF. Mo doping promotes the formation of oxygen vacancies, which optimizes the lattice structure and facilitates oxygen ion transport. In addition, Mo incorporation improves the charge transfer kinetics and overall electrical conductivity, with the PBSCFM01 sample exhibiting a maximum conductivity of 1051 S/cm at 250 °C—about 15% higher than that of pure PBSCF. Density functional theory calculations were further performed to elucidate the role of Mo incorporation in modulating oxygen vacancy formation. The theoretical results are in excellent agreement with the experimental observations, providing atomic-scale insight into the enhanced cathodic activity. This work demonstrates Mo as effective dopant for layered double perovskite materials to develop high oxygen reduction reaction activity cathode material PCFCs.

## Graphical abstract

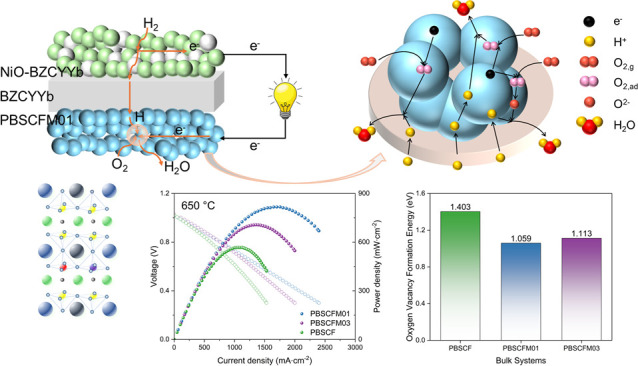

## 1 Introduction

The escalation of greenhouse gas emissions and environmental pollutants due to the excessive consumption of fossil fuels results in high attention to sustainable energy systems.^1,2^ One promising alternative among the emerging solutions is the synergistic utilization of green hydrogen^3,4^ produced from renewable sources such as solar and wind power, coupled with electrochemical energy conversion devices like fuel cells.^5–10^ Among them, solid oxide fuel cells (SOFCs) are distinguished by their superior efficiency in converting chemical energy to electricity.^11–13^ However, its wider adaptation remains hindered by durability concerns and cost challenges due to the elevated high operating temperature (800–1000 °C), which promotes detrimental effects such as material degradation, increased system maintenance costs, sealing challenges, etc.^14^ There has been more interest in protonic ceramic fuel cells (PCFCs) than SOFCs currently because of their enormous potential to operate at lower temperatures and, hence, overcome the main obstacles of SOFC commercialization. On the other hand, lower operating temperatures pose a challenge to the performance of the cathode. Since the cathode is the most critical part affecting the overall performance of the fuel cell, and the polarization resistance (*R*_p_) of the fuel cell is largely governed by the catalytic activity of the cathode, it becomes imperative to design and develop high-performing cathodes with superior catalytic activity for oxygen reduction reaction (ORR), high conductivity, and low *R*_p_, etc. to enhance the performance of PCFCs at the relatively lower operating temperatures. Therefore, recent efforts have been put into developing triple (electronic, ionic, and protonic) conducting oxides as cathode materials. Unlike traditional pure electronic conducting cathodes, triple conducting cathodes extend ORR active sites across the entire cathode surface, which effectively facilitates kinetics for ORR and enhances electrochemical performance. It is noteworthy that the initially identified mixed ionic and electronic conducting (MIEC) cathodes, such as Ba_0.5_Sr_0.5_Co_0.8_Fe_0.2_O_3‑δ_ (BSCF),^15^ PrBa_0.5_Sr_0.5_Co_1.5_Fe_0.5_O_5+δ_ (PBSCF),^16–18^ and BaCo_0.4_Fe_0.4_Zr_0.1_Y_0.1_O_3‑δ_ (BCFZY),^19^ have subsequently been found to exhibit triple conductivity and demonstrate excellent performance in PCFCs. Choi et al.^16^ initially reported PBSCF, which was found triple conducting and demonstrated outstanding performance with peak power density (PPD) of ∼2200 mW·cm^–2^ at 600 °C in SOFCs; they further extended using PBSCF in PCFCs alongside a chemically stable electrolyte, BaZr_0.4_Ce_0.4_Y_0.1_Yb_0.1_O_3+δ_ (BZCYYb4411), and the PPD of the anode-supported fuel cells surpassed 500 mW·cm^–2^ at 500 °C in wet air.^17^

As a high-valence transition element, doping molybdenum (Mo) was found to improve the lattice stability and due to its smaller ionic radii compared to other transition elements such as cobalt and iron, Mo doping can mitigate thermal expansion.^20–22^ However, the impact of Mo doping on conductivity, oxygen vacancy formation, and electrochemical performance are highly dependent on the structure and composition of the materials. Mo doping was found to reduce electrochemical performance,^20^ while other researchers reported that it enhanced hydration and proton migration ability, thereby significantly increasing power output when doping in BSCF.^21^ Similar findings appeared in Mo-doped La_0.5_Sr_0.5_FeO_3‑δ_, where the presence of Mo increased more oxygen vacancies toward ORR and improved the cathode performance.^22^ Therefore, the impact of Mo doping varies across different materials and requires broader investigation to optimize its doping strategy in order to enhance cathode performance.

Although Mo doping has been investigated in several perovskite-based cathodes, its influence is strongly dependent on the parent lattice, B-site chemistry, and operating environment. Therefore, the role of Mo cannot be directly generalized across different perovskite systems. In particular, the effect of B-site Mo-substitution in the layered double perovskite such as PrBa_0.5_Sr_0.5_Co_1.5_Fe_0.5_O_5+δ_ cathode for PCFC operation has not been systematically clarified. Therefore, establishing how Mo content regulates phase stability, oxygen-defect chemistry, charge transport, and cathodic reaction kinetics in PBSCF is essential for rationally optimizing this class of triple conducting cathodes. Therefore, in this work, a series of Fe-site Mo-doped PrBa_0.5_Sr_0.5_Co_1.5_Fe_0.5_O_5+δ_ cathode materials, denoted as PBSCFM_*x*_ (*x* = 0, 0.01, 0.03, and 0.05), were designed to clarify the composition-dependent role of Mo incorporation in layered PBSCF. The nonmonotonic effect of Mo doping on the phase structure, oxidation states, electrochemical performance, and electrical conductivity was investigated in detail. Furthermore, by combining comprehensive experimental characterization with density functional theory (DFT) calculations, we elucidate the atomic-scale mechanism by which Mo lowers oxygen vacancy formation energy and accelerates ORR kinetics, which provides mechanistic insights into designing the optimum dopant concentration required for tuning performance and achieving reliable cathodes for PCFCs.

## 2 Experimental Section

### 2.1 Cathode Powder Preparation

PrBa_0.5_Sr_0.5_Co_1.5_Fe_0.5_O_5+δ_ (PBSCF), PrBa_0.5_Sr_0.5_Co_1.5_Fe_0.49_Mo_0.01_O_5+δ_ (*x* = 0.01) (PBSCFM01), PrBa_0.5_Sr_0.5_Co_1.5_Fe_0.47_Mo_0.03_O_5+δ_ (*x* = 0.03) (PBSCFM03), and PrBa_0.5_Sr_0.5_Co_1.5_Fe_0.45_Mo_0.05_O_5+δ_ (*x* = 0.05) (PBSCFM05) powders were synthesized by the Pechini method. The precursor nitrates, including Pr­(NO_3_)_3_·6H_2_O (99.9%, Sigma-Aldrich), Ba­(NO_3_)_2_ (98%, Sigma-Aldrich), Sr­(NO_3_)_2_ (99%, Sigma-Aldrich), Co­(NO_3_)_3_·6H_2_O (99%, Sigma-Aldrich), Fe­(NO_3_)_3_·9H_2_O (99%, Sigma-Aldrich), and (NH_4_)_6_Mo_7_O_24_·4H_2_O (99%, Sigma-Aldrich) with the exact stoichiometric ratios, were fully dissolved in deionized water to form a clear solution. Citric acid (CA) and ethylene glycol (EG) were then added as complexing agents by the molar ratio of total metal ions, CA, EG of 1:2:3. When CA and EG were completely dissolved, the solution was heated to 300 °C under continuous stirring. After the solution was transformed from viscous colorful gel to black complex, the solid precursor was formed. Further, it was calcined in the furnace at 950 °C in air for 4 h to obtain final product with perovskite structure. The proton conducting electrolyte powder BaZr_0.1_Ce_0.7_Y_0.1_Yb_0.1_O_3‑δ_ (BZCYYb) and the ionic conducting electrolyte powder Gd_0.1_Ce_0.9_O_2‑δ_ (GDC) (Fuelcellmaterials) were used for the chemical compatibility test with PBSCFM03. BaZr_0.1_Ce_0.7_Y_0.1_Yb_0.1_O_3‑δ_ (BZCYYb) powder was synthesized via a conventional solid-state reaction method. Stoichiometric amounts of BaCO_3_, ZrO_2_, CeO_2_, Y_2_O_3_, and Yb_2_O_3_ were used as starting materials. The precursors were thoroughly mixed and ball-milled in ethanol for 12 h using zirconia balls, followed by drying and calcination at 1100 °C for 10 h to initiate phase formation. The calcined powders were subsequently remilled and calcined again at 1450 °C for 5 h in air to obtain single-phase BZCYYb powder. The obtained powders were ground before X-ray diffraction (XRD) characterization. The XRD spectra of BZCYYb are presented in Figure S1.

### 2.2 Cell Fabrication

To prepare electrolyte pellets for symmetrical cells, 0.4 g of GDC powder was dry-pressed under the pressure of 5 MPa to obtain the electrolyte disk with a thickness of approximately 0.5 mm. The GDC disks were further sintered in air at 1400 °C for 6 h and the dense GDC electrolyte pellets were formed. The cathode slurries were prepared by ball-milling synthesized PBSCFM_*x*_ powders with ink vehicle at a mass ratio of 1:0.8 over 24 h. Further, the slurry was uniformly screen-printed on both sides of the GDC pellets (circular radius of 3 mm) and then sintered at 950 °C for 4 h. Finally, both cathode surfaces were evenly applied with silver paste and connected silver wires, which play the role of a current collector. Therefore, symmetrical cells with the PBSCFM_*x*_ |GDC| PBSCFM_*x*_ configuration were meticulously fabricated for precise electrochemical impedance spectroscopy (EIS) measurements. Single cells with a configuration of NiO-BZCYYb |BZCYYb| PBSCFM_*x*_ were fabricated via a dry-pressing and drop-coating process. The NiO-BZCYYb anode substrate was prepared by mixing NiO and BZCYYb powders (60:40 wt %) in ethanol, followed by ball-milling for 12 h, drying, and presintering at 800 °C for 5 h in air. A dense BZCYYb electrolyte layer was subsequently deposited onto the presintered anode support by drop-coating BZCYYb suspension and sintered at 1400 °C for 5 h to achieve full densification. Finally, the PBSCFM_*x*_ cathode slurry was screen-printed onto the electrolyte surface and sintered at 950 °C for 4 h to complete the single-cell fabrication.

### 2.3 Physicochemical Characterizations

The phase structure of PBSCFM_*x*_ (*x* = 0, 0.01, 0.03, and 0.05) was investigated by XRD (PANalytical Empyrean XRD) utilizing a copper target Kα (α = 1.5418) with 40 kV working voltage and 40 mA working current. Rietveld refinement was conducted using BIOVIA Materials Studio software to provide insights into the materials’ crystal structure and lattice parameters. The morphology of cathode powders and the cross-sectional structure of symmetrical cells were inspected by scanning electron microscopy (SEM, Quanta 250 FEG ESEM) and high-resolution transmission electron microscopy (TEM, Jem-2100F Jeol). Energy-dispersive X-ray (EDX) mapping was conducted on Oxford Instruments X-Max 80T. X-ray photoelectron spectroscopy (XPS), using a Thermo Fisher Scientific ESCALAB Xi + system featuring a monochromatic Al Kα X-ray source (1486.6 eV), enabled accurate measurement of binding energies (BEs) and chemical states of their elements in cathode samples. Thermogravimetric analysis (TGA) conducted on NETZSCH STA 449 F3 Jupiter measured the mass change of cathode samples as a result of temperature in air, providing insights into the oxidation and reduction behavior through weight changes associated with oxygen loss or uptake. O_2_-temperature-programmed desorption (O_2_-TPD) measurements were carried out using a Micromeritics AutoChem III chemisorption analyzer to evaluate the oxygen desorption behavior of the cathode powders. For each measurement, approximately 0.15 mg of the sample was loaded into a quartz reactor. The sample was exposed to the O_2_/He mixture, 20 cm^3^ STP/min for 40 min to achieve oxygen adsorption, followed by purging with He to eliminate physically adsorbed oxygen. The desorption profile was then recorded by heating the sample from ambient temperature to 900 °C at a rate of 10 °C/min under flowing He, while the evolved oxygen species were continuously monitored as a function of temperature.

### 2.4 Electrochemical Characterizations

EIS is a commonly used method for probing the electrochemical processes such as charge transport, diffusion of ions, and surface reactions, making it ideal for characterizing fuel cell components—electrode and electrolyte. EIS measurements were carried out on a BioLogic VSP-3e electrochemical workstation connecting symmetrical cells, covering a frequency range from 100 kHz to 0.1 Hz at an AC perturbation amplitude of 30 mV under open-circuit conditions. Symmetrical cell EIS measurements were conducted in dry air from 500 to 650 °C. The measurements began at 650 °C and were performed with a 50 °C decrement until reaching 500 °C. The resulting data were then analyzed and fitted to equivalent circuit models using ZView software. Before conducting conductivity measurements, the cathode powders were pelletized into dense cuboid bars and sintered at 1200 °C in air for 10 h. A four-point probe method is used for measurement as it offers more accurate and reliable results than the two-point probe method, which uses one pair of electrodes to apply the current and a separate pair to measure the voltage. For single cell testing, 3 vol %H_2_O-humidified H_2_ was supplied to the anode, and dry ambient air was supplied to the cathode. The gas flow rates were 80 SCCM for humidified H_2_.

### 2.5 Density Functional Theory Calculations

DFT were carried out using the plane wave-based Vienna ab Initio Simulation Package (VASP).^23^ Spin-polarized calculations with a dispersion correction method (DFT-D3) were performed in the systems. Long-range dispersion forces of van der Waals interactions were obtained using Grimme’s method.^24^ The generalized gradient approximation with the Perdew–Burke–Ernzerhof functional (GGA-PBE) was employed to describe the exchange–correlation energy.^25,26^ Electron–ion interactions were described using the projector augmented wave method.^27^ An energy cutoff of 520 eV for the plane-wave basis set was used for the convergence of the total energy. The convergence criteria were set to 10^–4^ eV and 0.02 eV/Å for the electronic self-consistent iteration and the forces on each atom, respectively, while the Γ-point sampling was employed for large supercell calculations. To construct a structural model close to the experimental composition while maintaining a reasonable computational cost, the PBSCF model was initially established by substituting half amount of Ba atoms with Sr atoms and quarter amount of Co atoms with Fe atoms to form supercells with 4 × 4 × 2 from the initial crystal structure of PrBaCo_2_O_6_ obtained from the Materials Project^28^ database. For Mo-doped systems, one Fe atom was substituted by Mo to represent the PBSCFM01 system, while two Fe atoms were substituted by Mo to construct the PBSCFM03 system, corresponding to different Mo doping concentrations within the same supercell.

Oxygen vacancies were introduced by removing one oxygen atom from the relaxed supercell. The oxygen vacancy formation energy was calculated according to the following expression

1 $$E_{f}=E_{defect}-E_{p\mathrm{erf}ect}+\frac{1}{2}E_{O_{2}}$$

where *E*_defect_ and *E*_perfect_ are the total energies of the supercell with and without an oxygen vacancy, respectively, and *E*_O2_ is the total energy of an isolated O_2_ molecule that calculated within the same DFT framework.

## 3 Results and Discussion

### 3.1 Physicochemical Characterizations for PBSCFM_*x*_

To study the effect of Mo doping on the phase structure of materials, four cathode materials PBSCFM_*x*_ (*x* = 0, 0.01, 0.03, and 0.05) of different Mo compositions were first examined by XRD. Figure 1a illustrates the XRD spectra of PBSCFM_*x*_ (*x* = 0, 0.01, 0.03, and 0.05). PBSCF, PBSCFM01, and PBSCFM03 show pure phases that correspond well to the characteristic peaks of PrBaCo_2_O_5+δ_ (PDF# 00-053-0131) and adopt a layered double perovskite structure with the space group of *P*4/*mmm*. However, as the Mo doping content increased to 5%, the impurity phase BaMoO_4_ was detected in PBSCFM05, as shown in Figure 1a, at a diffraction angle of ∼27.2°. Quantitative phase analysis using a two-phase Rietveld refinement model shows that PBSCFM05 consists of 2.35 wt % BaMoO_4_ impurity, as shown in Figure S2. This result indicated that the maximum amount of Mo-substitution at the Fe site in PBSCF lattice under the present synthesis conditions is 3%. This finding indicates that the beneficial role of Mo is not simply proportional to its nominal content but is constrained by the phase stability of the PBSCF host lattice. Therefore, the following physicochemical and electrochemical analyses focus on the phase-pure PBSCF, PBSCFM01, and PBSCFM03 compositions to clarify how lattice-incorporated Mo, rather than secondary Mo-containing phases, affects oxygen-defect behavior and cathodic activity. Figure 1b further shows zoomed-in XRD spectra of three pure samples. It can be observed that doping Mo into PBSCF resulted in a subtle shift to the higher diffraction angle in the peak positions as the Mo content increased. This shift indicates lattice parameter changes due to the substitution of Mo for Fe. Since Mo and Fe cations have different ionic radii, replacing Fe with Mo alters the unit cell dimensions, leading to shifts in the diffraction angles. To improve the accuracy of the XRD analysis, Rietveld refinements for PBSCF, PBSCFM01, and PBSCFM03 were performed, and the corresponding results are presented in Figure 1c and Table 1. The low values of *R*_wp_ (weighted profile residual factor) and *R*_p_ (profile residual factor) indicate a statistically good fitting, meaning the refinement accurately models the experimental diffraction data. The crystal structure of PBSCFM01 is defined in the *P*4/*mmm* space group and showed lattice parameters of *a* = *b* = 3.88 Å and *c* = 7.76 Å, whereas the lattice parameters of PBSCFM03 are *a* = *b* = 3.87 Å and *c* = 7.75 Å, which further verified that substituting Mo cations with smaller ionic radii (0.59 Å) to Fe cations with larger ionic radii (0.65 Å)^29^ led to a decrement in the lattice volume due to the more compact arrangement of atoms.

**Figure 1:**
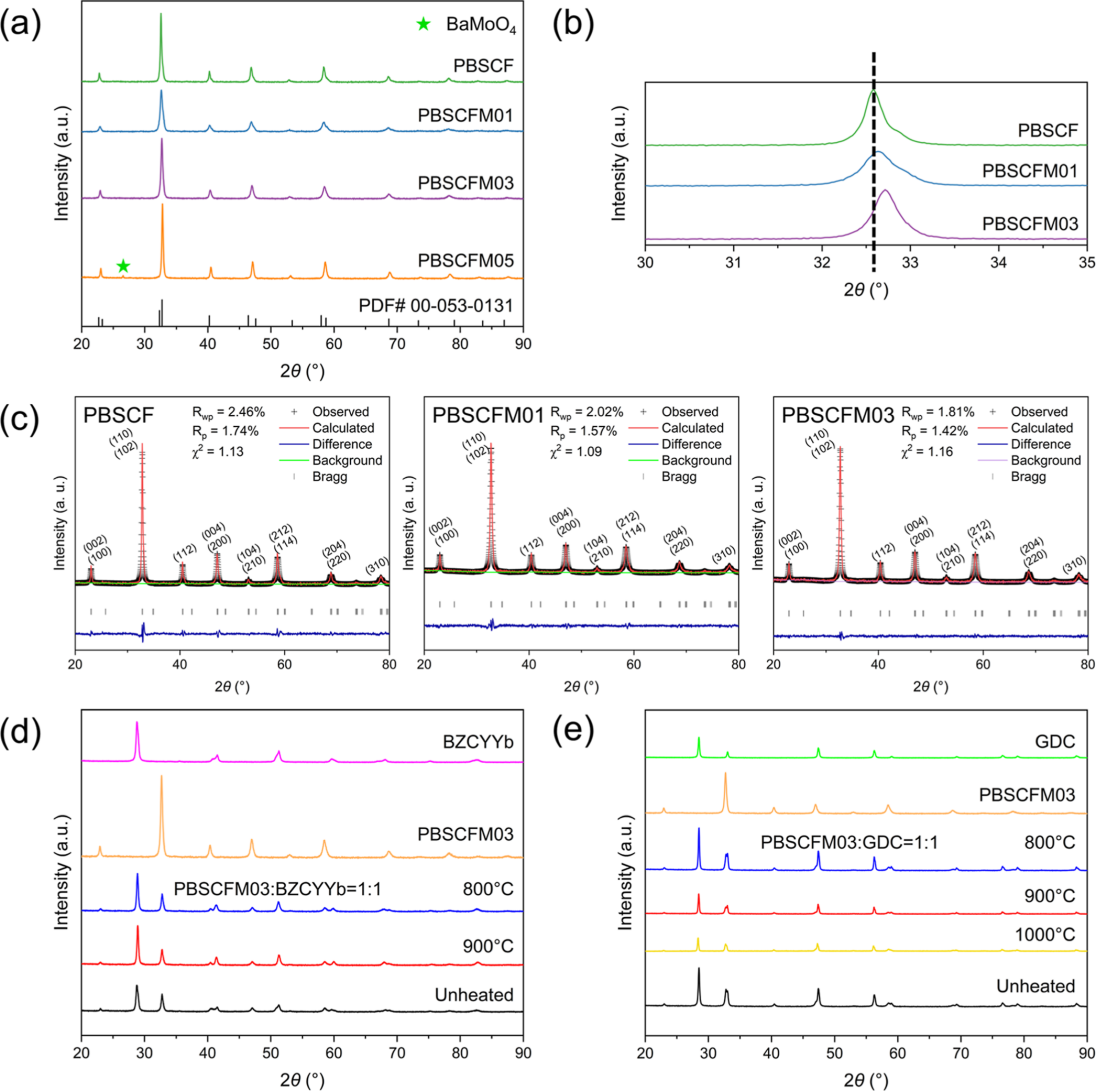
(a) XRD patterns of PBSCFM_*x*_ (*x* = 0, 0.01, 0.03, and 0.05) calcined at 950 °C; (b) zoomed-in XRD patterns of PBSCFM_*x*_ (*x* = 0, 0.01, and 0.03); (c) Rietveld refinement results of PBSCF, PBSCFM01, and PBSCFM03; (d) XRD patterns of the PBSCFM03-BZCYYb mixture for the chemical compatibility test; (e) XRD patterns of the PBSCFM03-GDC mixture for the chemical compatibility test.

**Table 1:** Rietveld Refinement Results for PBSCFM_*x*_ (*x* = 0, 0.01, and 0.03)

samples	space group	*a* (Å)	*c* (Å)	*V*(Å^3^)	*R*_wp_(%)	*R*_p_(%)
PBSCF	*P*4/*mmm*	3.89	7.77	117.71	2.46	1.74
PBSCFM01	*P*4/*mmm*	3.88	7.76	117.05	2.02	1.57
PBSCFM03	*P*4/*mmm*	3.87	7.75	116.24	1.81	1.42

PBSCFM03 was selected for the chemical compatibility evaluation because it represents the highest Mo-doped composition that maintains a single-phase layered double perovskite structure under the present synthesis conditions, whereas further Mo incorporation in PBSCFM05 leads to BaMoO_4_ impurity formation; therefore, PBSCFM03 provides the strictest phase-pure case for assessing possible reactions with GDC and BZCYYb electrolytes. To assess the chemical compatibility between PBSCFM_*x*_ cathodes and GDC or BZCYYb electrolytes, the phase stability was accessed by mixing powders of PBSCFM03-GDC and PBSCFM03-BZCYYb in the weight ratio of 1:1 and calcined at various temperatures. Figure 1d,e presents XRD spectra of PBSCFM03-BZCYYb and PBSCFM03-GDC mixtures. The observed diffraction peaks correspond exclusively to the PBSCFM03 and BZCYYb (or GDC) phases within the composite powders without additional impurity peaks detected. This indicates robust chemical compatibility between PBSCFM03 and these two electrolytes, indicating that no secondary reactions occur during the preparation or testing.

Figure 2a illustrates TEM image of PBSCFM01 powder calcined in air at 950 °C. High-resolution TEM (HRTEM) image shows well-defined lattice fringes across large domains, evidencing good crystallinity of PBSCFM01. It reveals that the (110) plane lattice fringe spacing values of PBSCFM01 is 2.64 Å, which is slightly lower than that of PBSCF (2.74 Å), as shown in Figure S3. This is consistent with the larger ionic radius of Fe against Mo as stated earlier. HAADF-STEM image and the corresponding EDX mapping of PBSCFM01 powder (Pr, Ba, Sr, Co, Fe, Mo, and O) in Figure 2c demonstrate a spatially uniform distribution of all cations and oxygen throughout the probed crystallite. Specifically, the Mo signal overlaps well with that of entire surface of PBSCF, suggesting that Mo is successfully incorporated into the B-site of the perovskite lattice by partially substituting Fe. Within the spatial resolution of the mapping, no elemental segregation, core–shell contrast, or surface enrichment is observed. The compositional homogeneity, together with the lattice ordering seen in HRTEM, is consistent with a single-phase, well-crystallized PBSCFM01. The TEM image and EDX mapping of PBSCFM03 are shown in Figure S4.

**Figure 2:**
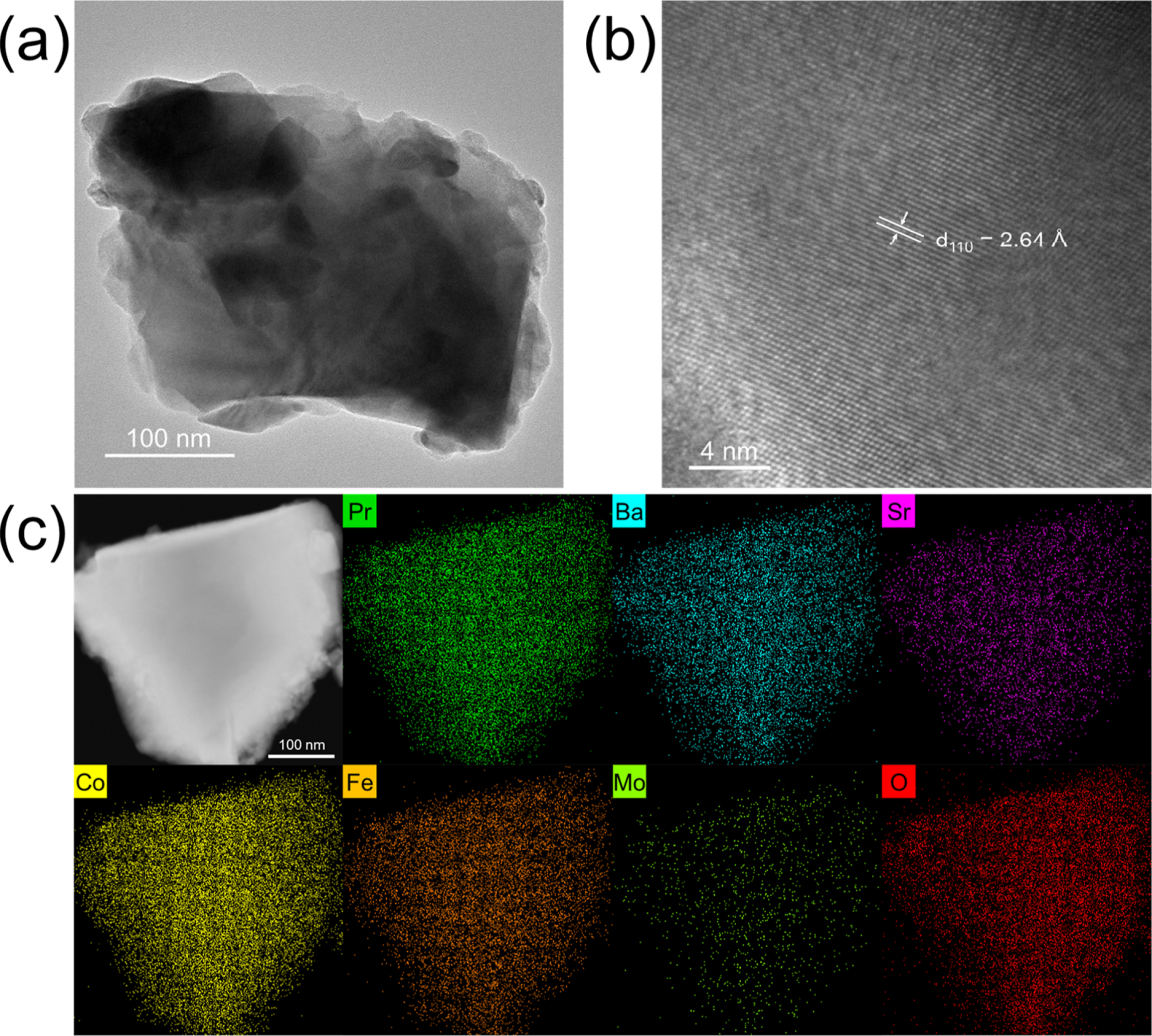
Microstructure characterization of the PBSCFM01 cathode. (a) TEM image of PBSCFM01 powder; (b) high-resolution TEM image of PBSCFM01 powder in (a); (c) EDX-mapping results of PBSCFM01.

The kinetics of ORR and charge transfer processes are predominantly influenced by the oxidation state of transition metals located at the B-site of the perovskite structure and the abundance of oxygen vacancies within the material.^30^ XPS was employed to investigate the oxidation states of constituent elements within the PBSCFM_*x*_ perovskite material. All XPS spectra deconvolutions presented in this paper were carried out according to the referenced literature.^31^ Figure 3a presents the O 1s XPS spectra of PBSCF, PBSCFM01, and PBSCFM03 samples, and the proportions of each species derived from the fitting are shown in Table 2. The O 1s spectra can be deconvoluted into different oxygen species, with peaks corresponding to adsorbed oxygen (O_ads_, such as O^–^ or O2–2), lattice oxygen (O_L_, mainly O^2–^), and adsorbed water (H_2_O_ads_). The BEs of O_L_ in PBSCF, PBSCFM01, and PBSCFM03 are 528.6, 529.5, and 529.4 eV, respectively. The higher BE of O_L_ indicates the weaker Coulombic force between O_L_ and B-site cations, which indicates that the mobility of lattice oxygen is increased^32,33^ in favor of the ORR activity of the cathodes of PBSCFM01 and PBSCFM03. With Mo doping, the proportion of O_ads_ in PBSCFM01 drastically increased from 44.75% (PBSCF without Mo doping) to 62.84%, and the ratio of O_ads_ to O_L_ (O_ads_/O_L_) increased from 1.915 to 2.183. The abundant adsorbed oxygen on the cathode surface promotes oxygen diffusion toward the cathode/electrolyte interface, thereby increasing the ORR activity in the cathode. This indicates the positive effect of Mo doping on ORR as O_ads_ is directly involved in surface catalytic processes as follows^34^

2 $$O_{2}\overset{e^{-}}{\to }O_{2}^{-}\overset{e^{-}}{\to }O_{2}^{2-}\to {2O}^{-}\overset{2e^{-}}{\to }2O^{2-}$$

More importantly, unlike lattice oxygen represents oxygen atoms that are integrated into the crystal lattice of the perovskite where oxygen is bonded to metal atoms in a well-defined structural arrangement, adsorbed oxygen corresponds to oxygen species that are physically or chemically adsorbed on the surface which are not part of the crystal structure. Oxygen vacancies, especially those located at or near the surface, can act as active sites for oxygen adsorption. Therefore, a higher concentration of oxygen vacancies might reflect increased O_ads_, which in return indicates that PBSCFM01 created more oxygen vacancies after incorporating Mo into its lattice structure. This is further verified by TGA results.

**Figure 3:**
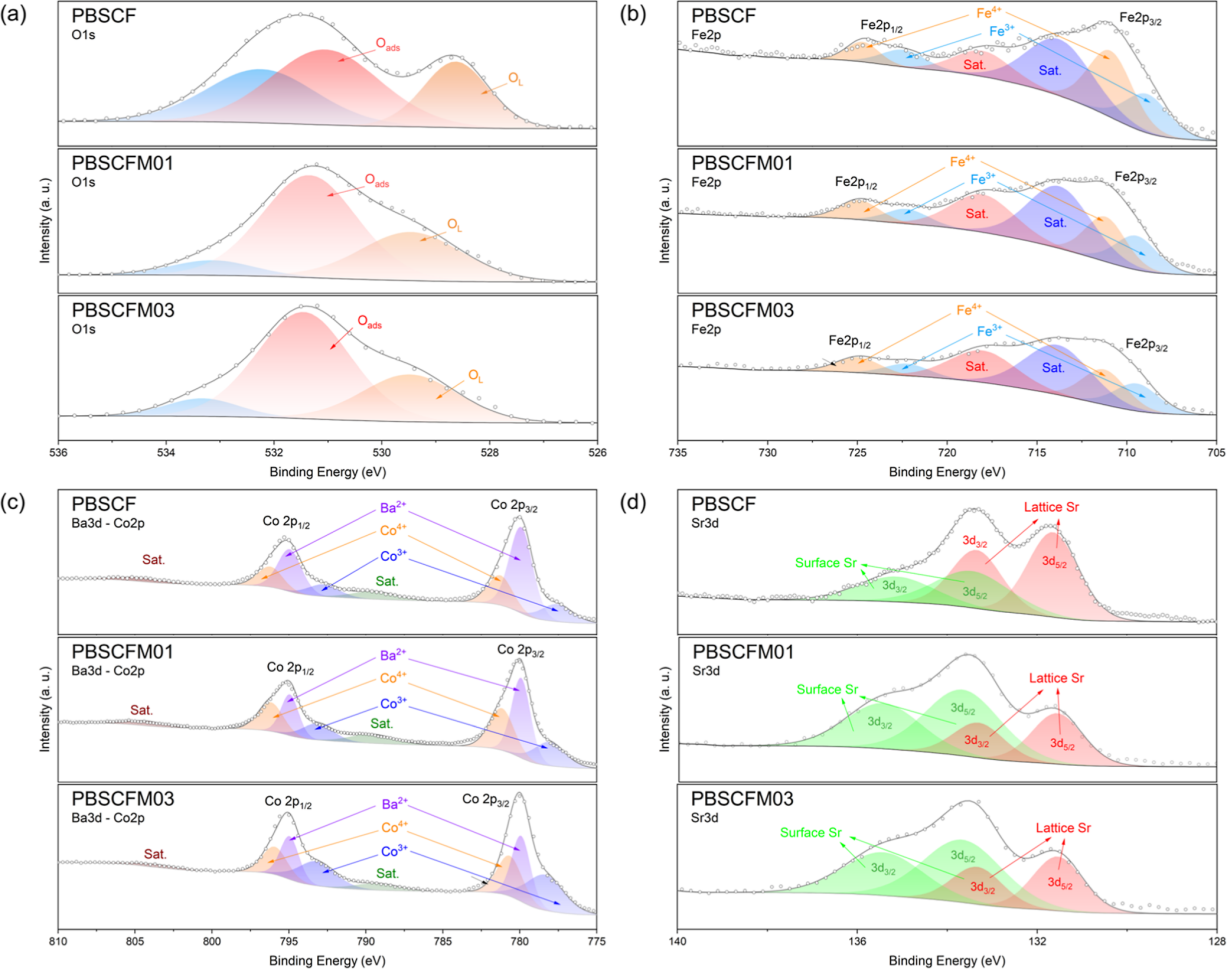
XPS spectra of (a) O 1s, (b) Fe 2p, and (c) Ba 3d-Co 2p, (d) Sr 3d for PBSCFM_*x*_ (*x* = 0, 0.01, and 0.03).

**Table 2:** Proportion of Each Species of O for PBSCF, PBSCFM01, and PBSCFM03 Samples

samples	proportion of O 1s (%)			O_ads_/O_L_
	lattice O	adsorbed O	H_2_O	
PBSCF	23.37	44.75	31.88	1.915
PBSCFM01	28.79	62.84	8.37	2.183
PBSCFM03	31.03	61.12	7.58	1.970

Besides, with increasing Mo doped into PBSCF, the lower ratio of O_ads_/O_L_ is observed in PBSCFM03 (1.970) to PBSCFM01 (2.183), which can be explained by the charge compensation mechanism and lattice structure stabilization. At low Mo doping (*x* = 0.01), introducing Mo^6+^ can modify the surface electronic structure or create local defects that enhance oxygen adsorption.^35^ At higher Mo contents (*x* = 0.03), the lattice adjusts to accommodate high-valence Mo^6+^ by reducing the number of oxygen vacancies. This can happen through charge compensation mechanisms, such as lowering oxidation states of B-site metal cations or filling oxygen vacancy sites, leading to a more-stoichiometric structure. This can be justified by Fe 2p and Ba 3d-Co 2p XPS spectra in Figure 3b,c. Table 3 provides a comprehensive summary of the proportion of oxidation states of Fe and Co. In addition, as Figure 3d and Table S2 elaborate the XPS spectra of Sr 3d for PBSCFM_*x*_ (*x* = 0, 0.01, and 0.03) and the proportion of each species of Sr derived from the fitting results, doping Mo from 0.01 to 0.03 stabilizes Sr in the lattice and prevents its segregation to the surface, which is critical for enhancing structural stability and long-term performance of the material in PCFC application. The XPS spectra of Pr 3d are shown in Figure S5. The Mo 3d XPS spectrum, shown in Figure S6, indicates that Mo exists predominantly in a high-valence state in the air-calcined PBSCFM_*x*_ samples. Although mixed Mo^5+^/Mo^6+^ states can occur in some perovskite oxides depending on the composition and atmosphere, Mo^6+^ is commonly observed as the dominant Mo valence under oxidizing conditions. Therefore, Mo is treated as predominantly Mo^6+^ in the charge-compensation discussion.^36,37^

**Table 3:** Proportion of Each Oxidation State of Fe and Co for PBSCF, PBSCFM01, and PBSCFM03

samples	proportion of Fe valence (%)		proportion of Co valence (%)	
	Fe^3+^	Fe^4+^	Co^3+^	Co^4+^
PBSCF	57.54	42.46	43.11	56.89
PBSCFM01	50.83	49.17	57.78	42.22
PBSCFM03	48.15	51.85	55.09	44.91

In perovskite oxides like PBSCF, the B-site is occupied by transition metals (Co and Fe), and their average oxidation state influences the oxygen vacancy concentration (δ). A lower average B-site valence typically means the transition metals are in lower oxidation states, which is compensated by a reduction in lattice oxygen (high oxygen vacancy) to maintain charge neutrality. According to Tables 3 and S1, the average oxidation states of B-site Co and Fe cations for each sample are +3.53, +3.45, and +3.50, respectively. These results align directionally with the adsorbed oxygen values aforesaid, which reflects that doping Mo can promote oxygen vacancy formation, and PBSCFM01 has the most oxygen vacancies among the three samples.

Although TGA cannot directly quantify oxygen vacancies, it can provide critical indirect information about oxygen vacancy by tracking weight changes due to oxygen loss or uptake. Figure 4a presents the weight variation of three cathode materials as a function of temperature in dry air from room temperature to 1000 °C. The initial weight loss (below ∼100 °C) is likely due to the loss of adsorbed water or surface hydroxyl groups, which are common in porous materials or those exposed to ambient air. From ∼300 °C onward, the weight loss continues steadily and likely corresponds to the loss of lattice oxygen as temperature increases. This oxygen release is associated with the formation of oxygen vacancies. PBSCFM01 and PBSCFM03 showed higher weight losses (1.87% and 1.78% compared to 1.61% of PBSCF). This trend again indicates that the introduction of Mo leads to increased oxygen release (and, consequently, more oxygen vacancies) at higher temperatures. Such an effect likely originates from alterations in the electronic configuration and the local bond environment within the perovskite lattice after substituting Mo for Fe. PBSCFM01 experienced the largest oxygen loss which indicates the most oxygen vacancies formed. This indicates the previously stated positive correlation between adsorbed oxygen from XPS of O 1s and oxygen vacancy from TGA results.

**Figure 4:**
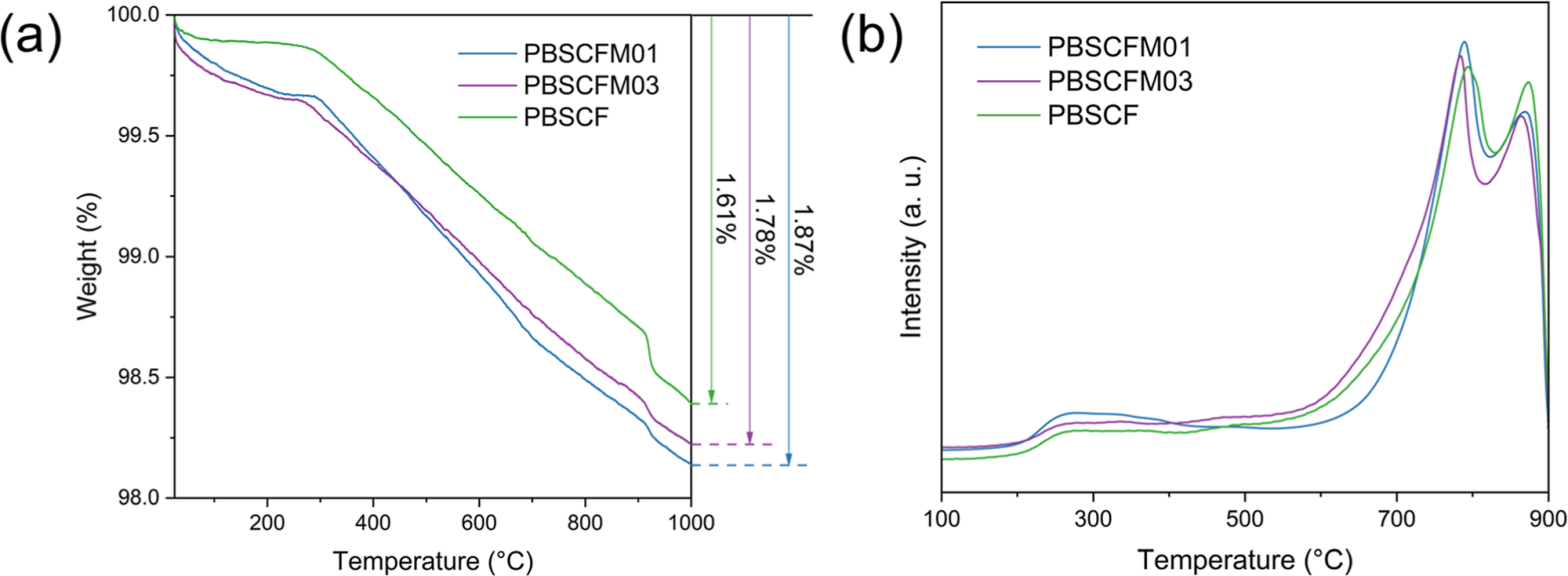
(a) TGA curves of PBSCFM_*x*_ (*x* = 0, 0.01, and 0.03) from RT to 1000 °C; (b) O_2_-temperature-programmed desorption (O_2_-TPD) profiles of PBSCFM_*x*_ (*x* = 0, 0.01, and 0.03).

O_2_-TPD was employed to probe the adsorption/desorption behavior of surface oxygen species and the lability of lattice oxygen of three cathode materials. The O_2_-TPD profiles of pristine PBSCF, PBSCFM01, and PBSCFM03 clearly show that Fe-site Mo incorporation alters the oxygen desorption behavior of the cathode. All samples exhibit a weak low-temperature desorption feature at approximately 220–350 °C, which can be assigned to weakly adsorbed surface oxygen species. This feature becomes more pronounced after Mo doping, especially for PBSCFM01, indicating that Mo-substitution increases the proportion of surface-active oxygen species, likely through the creation of additional oxygen-deficient sites. More significant differences appear in the high-temperature region above ∼600 °C, where desorption is mainly associated with lattice oxygen. Compared with pristine PBSCF, PBSCFM03 shows an earlier onset of oxygen release and a stronger desorption signal in the 650–750 °C range, suggesting that Mo doping weakens oxygen binding and facilitates oxygen migration within the lattice. PBSCFM01 exhibits the highest principal desorption peak at around 780–800 °C, whereas pristine PBSCF retains a more pronounced second high-temperature peak near 860–880 °C, indicative of a larger fraction of more strongly bound lattice oxygen. These results suggest that Mo-substitution does not simply increase the desorption intensity monotonically, but rather redistributes the oxygen species from strongly bound states toward more labile oxygen that can be released at lower temperature.

The O_2_-TPD results are fully consistent with the XPS and TGA results, showing an increase in oxygen vacancy concentration after Mo doping. In particular, the enhanced low-temperature desorption suggests that Mo doping increases the proportion of vacancy-associated active oxygen species and promotes vacancy-mediated oxygen transport. From the perspective of the PCFC cathode function, such changes are highly beneficial because they can accelerate oxygen adsorption/dissociation and improve the oxygen surface exchange process. Among the three compositions, PBSCFM01 shows the strongest main desorption response, implying a comparatively large reservoir of the releasable lattice oxygen. Overall, the O_2_-TPD results support the conclusion that moderate Mo-substitution is an effective strategy to activate the oxygen sublattice of PBSCF and, thereby, enhance its cathodic reaction kinetics.

To achieve a deeper understanding of the influence of Mo doping on oxygen vacancy formation, oxygen vacancy formation energy was evaluated based on DFT calculations. The structural stability of pristine PBSCF and two Mo-doped bulk systems were first investigated as shown in Figure 5a-c. The results indicate that configurations in which Mo dopants are spatially well separated are energetically more favorable than those with closely located Mo atoms, suggesting a tendency for Mo dopants to avoid clustering in the bulk lattice.

**Figure 5:**
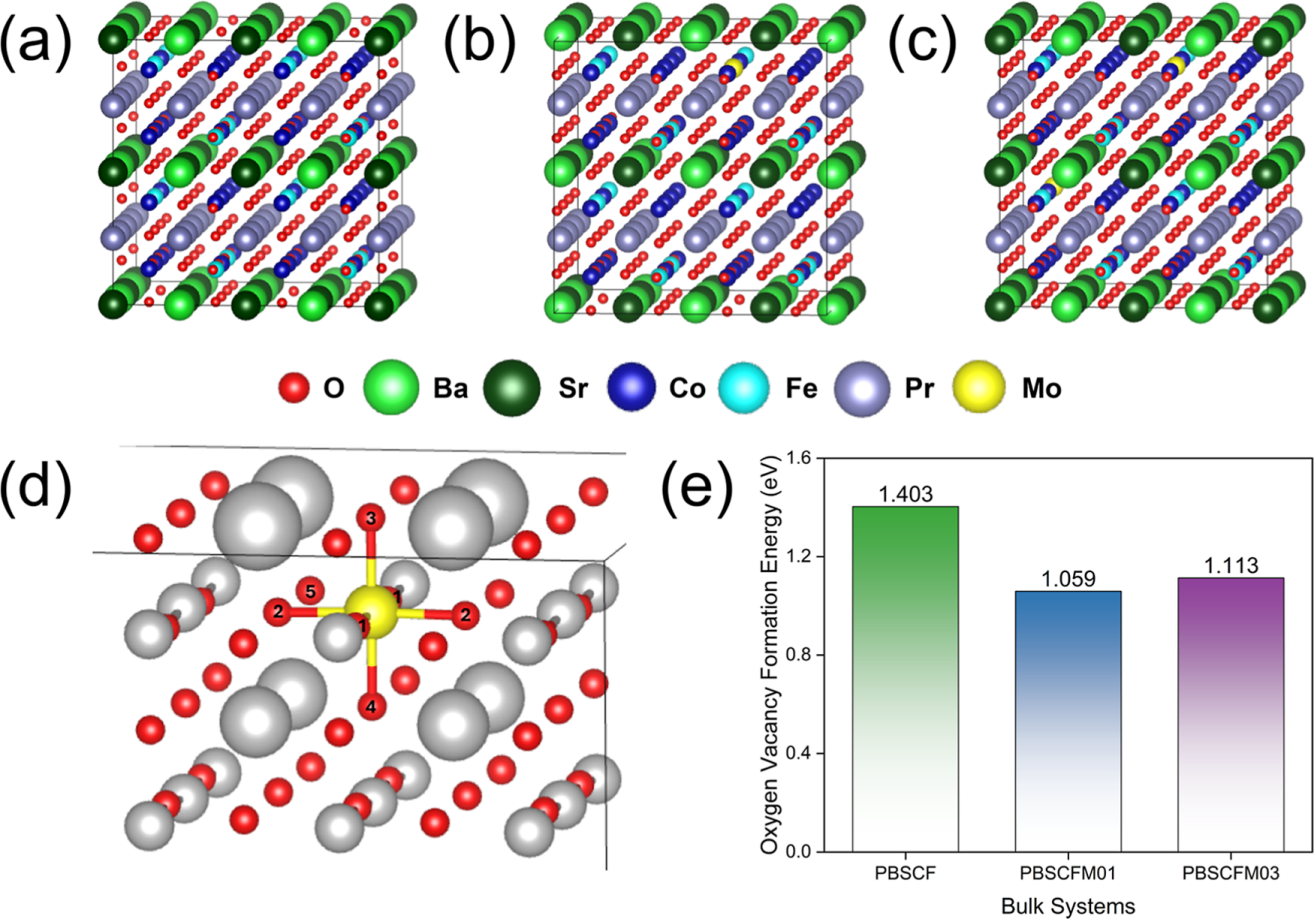
Optimized crystal structures with the lowest total energies for (a) pristine PBSCF, (b) PBSCFM01, and (c) PBSCFM03 bulk systems. For PBSCFM03, six different doping configurations were examined, and the most stable configuration with the lowest total energy is shown in (c). (d) Schematic illustration of the five distinct oxygen vacancy sites considered in this work, atoms of all elements except Mo and O are shown in uniform color for simplicity. (e) The lowest values of oxygen vacancy formation energy in pristine PBSCF, PBSCFM01, and PBSCFM03 bulk systems among five oxygen vacancy sites.

Moreover, the oxygen vacancy formation energy of both PBSCFM01 and PBSCFM03 systems is significantly reduced, indicating that Mo doping has a pronounced effect on promoting the formation of oxygen vacancies in the bulk system, as presented in Figure 5d,e. Notably, the introduction of a single Mo atom significantly promotes oxygen vacancies, and further increasing the Mo content leads to a diminished enhancement. This behavior suggests that the primary contribution to vacancy stabilization arises from the initial modification of the local electronic structure induced by Mo-substitution, whereas additional Mo dopants introduce competing effects, such as increased lattice distortion or dopant–dopant interactions, which partially offset the vacancy promising effect. Therefore, these DFT results offer a microscopic explanation for the experimentally observed effect of the Mo doping.

To quantitatively evaluate the oxygen nonstoichiometry, iodometric titration was performed for PBSCF, PBSCFM01, and PBSCFM03. The room-temperature oxygen contents were determined to be 5.984, 5.990, and 5.965 mol O per formula unit, respectively, as shown in Table S3. These values were further used as reference points to convert the TGA weight-loss curves into temperature-dependent oxygen contents. The converted oxygen content curves in Figure S7 show that the Mo-doped samples possess lower oxygen contents than pristine PBSCF in the elevated-temperature region, indicating enhanced thermally activated oxygen vacancy formation after Mo incorporation. At 650 °C, the oxygen contents of PBSCF, PBSCFM01, and PBSCFM03 are 5.741, 5.656, and 5.636 mol O per formula unit, respectively. The higher oxygen deficiency of the Mo-doped samples is consistent with the O_2_-TPD and DFT results, confirming that Mo-substitution facilitates oxygen release and oxygen vacancy formation.

### 3.2 Electrochemical Characterizations of PBSCFM_*x*_

To assess the *R*_p_ of the PBSCFM_*x*_ cathodes, symmetrical cells were fabricated using GDC as the electrolyte. GDC was used as the electrolyte for symmetrical cell EIS measurements for comparative evaluation of cathodic polarization behavior. This configuration allows the intrinsic differences among PBSCF, PBSCFM01, and PBSCFM03 cathodes to be screened under identical testing conditions. Moreover, the GDC-based symmetrical cell results are interpreted mainly as comparative cathode-screening data, mainly reflecting oxygen reduction and oxygen surface exchange with minimal additional complexities associated with proton conduction, hydration, and water-related processes in proton-conducting electrolytes. The cross-sectional morphology of the symmetrical cell of PBSCFM01 is presented in Figure 6a, revealing that the PBSCFM01 cathode layer possesses an approximate thickness of ∼23 μm with uniform porosity, which is beneficial for oxygen diffusion and ORR. Moreover, the GDC electrolyte is notably dense and exhibits excellent adhesion to the cathode. The cross-sectional morphology of the symmetrical cell of PBSCFM03 is presented in Figure S8. The electrochemical processes occurring at the cathode can be effectively investigated by analyzing EIS measured under various temperatures. Figure 6b shows the Nyquist plots that present EIS results of PBSCFM_*x*_ (*x* = 0, 0.01, and 0.03) cathodes at the temperature of 650 °C, 600 °C, 550 °C, and 500 °C, respectively. Table S4 illustrates the *R*_p_ results of three cathodes within the temperature range of 500–650 °C. The value of *R*_p_ can be determined by locating the intercept on the real impedance axis of the Nyquist plot, which corresponds to the intersection point of the low-frequency and high-frequency arcs.

**Figure 6:**
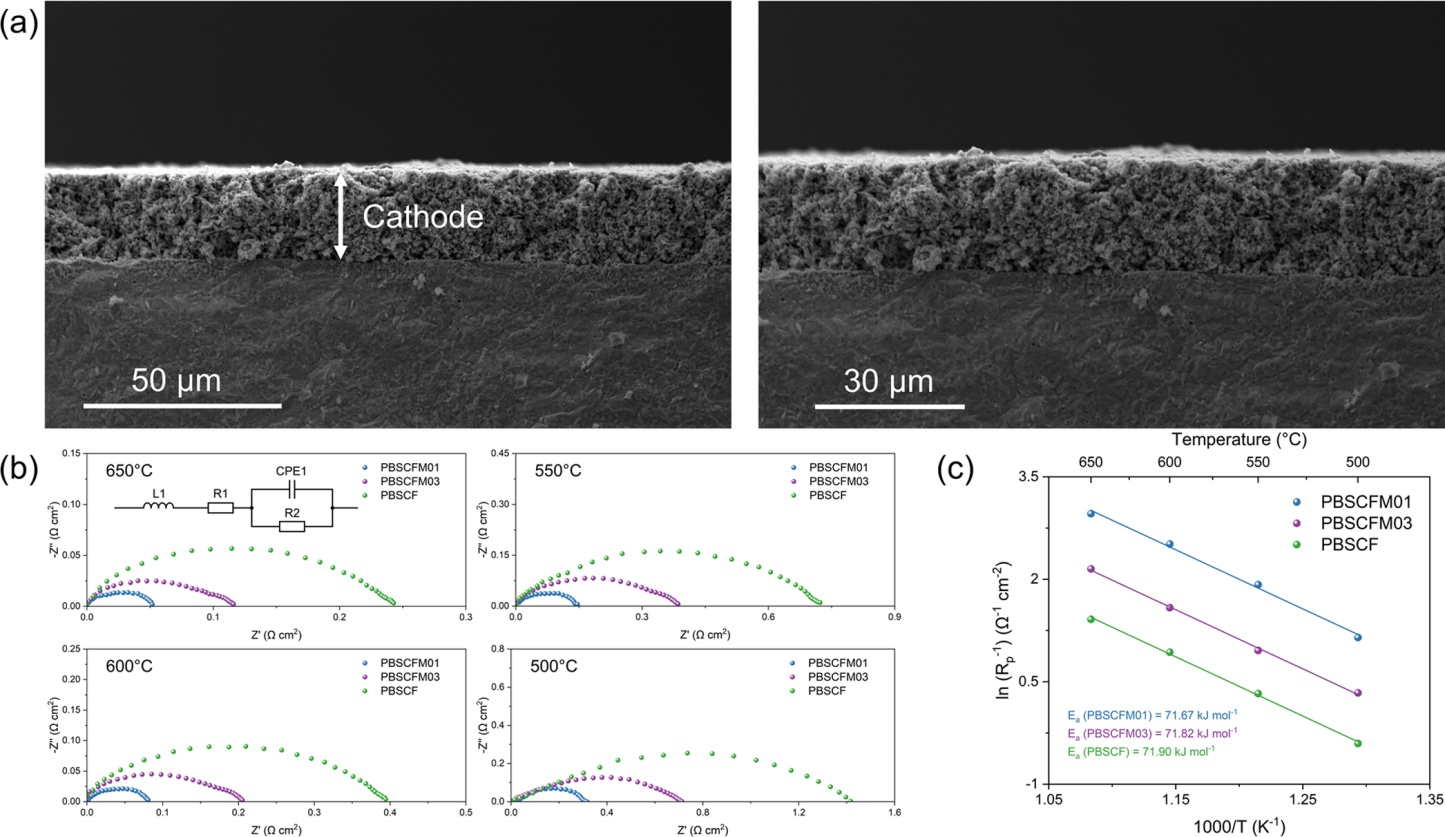
(a) SEM image of the cross-sectional PBSCFM01 |GDC| PBSCFM01 symmetrical cell; (b) EIS results of PBSCFM_*x*_ (*x* = 0, 0.01, and 0.03) cathodes on GDC-based symmetric cells at 650 °C, 600 °C, 550 °C, and 500 °C; (c) the Arrhenius plot of *R*_p_ of PBSCFM_*x*_ (*x* = 0, 0.01, and 0.03) on GDC-based symmetric cells between 500 and 650 °C in dry air.

All EIS results were fitted using a L_ohm_-R_E1_-(R_E2_-CPE1) equivalent circuit model, as depicted in Figure 6b. As shown in Figure 6b, the doping concentration of Mo has a profound influence on the ORR activity. The *R*_p_ of PBSCFM_*x*_ cathodes on the GDC electrolyte are 0.242, 0.052, and 0.116 Ω·cm^2^ for PBSCF, PBSCFM01, and PBSCFM03 cathodes at 650 °C, respectively. As it has been demonstrated that catalysts with high electrical conductivity exhibit lower charge transfer resistance,^38,39^ The *R*_p_ of PBSCFM01 and PBSCFM03 cathodes with higher conductivity are lower than that of the PBSCF cathode across all temperature ranges. The enhanced catalytic activity is likely attributable to the incorporation of Mo^6+^ into the Fe^3+^/Fe^4+^, which effectively increases oxygen vacancies concentration, thereby promoting oxygen adsorption and subsequent electron transfer that facilitates the formation of O^–^ species in the ORR. With an excessive Mo doping concentration (PBSCFM03), the *R*_p_ decreased relative to that of PBSCFM01, which is consistent with the previously observed trends in oxygen vacancy concentration and electrical conductivity. This phenomenon can be attributed to the reduced concentration of oxygen vacancies resulting from excessive Mo doping. In addition, the temperature dependence of *R*_p_ for each cathode is displayed in the Arrhenius plot in Figure 6c. The activation energy (*E*_a_), calculated from the fitting, are 71.90, 71.67, and 71.82 kJ/mol for PBSCF, PBSCFM01, and PBSCFM03 cathodes, respectively. PBSCFM01 and PBSCFM03 exhibit lower *E*_a_ compared to PBSCF, revealing that the partial doping of Mo at the Fe site in PBSCF results in a decrease in the *E*_a_, with PBSCFM01 exhibiting the lowest *E*_a_ among the three cathodes. The lower *E*_a_ implies that PBSCFM01 maintains more stable performance at lower temperatures (typically below 400 °C) relative to materials with higher *E*_a_.^40^

Furthermore, a distribution of relaxation times (DRT) investigation was conducted to deconvolute the elementary processes involved in the ORR. This powerful tool enables more detailed information on individual electrochemical relaxation processes by analyzing EIS results.^41^ As illustrated in Figure 7a, the DRT plot exhibits three well-separated peaks, denoted as LF, IF, and HF. The low-frequency (LF) region is generally linked to diffusion of oxygen gas across the cathode surface. The intermediate-frequency (IF) peak primarily reflects the surface exchange reaction and bulk charge transfer processes. The high-frequency (HF) peak corresponds to rapid charge-transfer processes occurring at the electrode–electrolyte interface, indicating intrinsic electrochemical reaction kinetics. In Figure 7a, PBSCF shows the largest LF and IF peaks, indicating that gas transport/surface exchange is the rate-limiting step for the undoped PBSCF cathode. Both Mo-doped cathodes, PBSCFM01 and PBSCFM03, display markedly suppressed LF and IF intensities and a slight right-shift of these peaks, evidencing faster oxygen surface exchange and reduced transport limitations. The HF features are weak for all three materials and comparable in magnitude, suggesting similar interfacial/bulk transport at short time scales. Overall, the integrated DRT response implies that PBSCFM01 delivers the lowest process-resolved *R*_p_ value and the most favorable ORR kinetics among the three cathodes.

**Figure 7:**
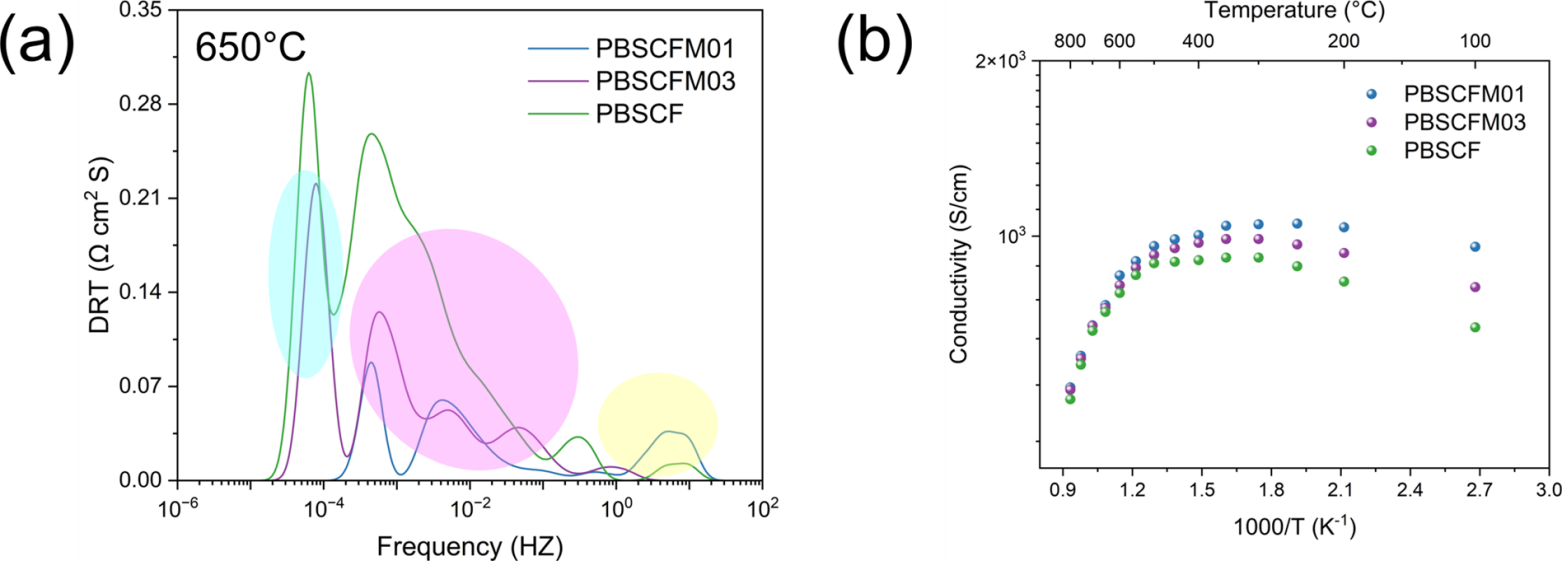
(a) Comparison of DRT plots of PBSCFM_*x*_ cathodes at 650 °C; (b) electrical conductivity of PBSCFM_*x*_ (*x* = 0, 0.01, and 0.03) from 100 to 800 °C in air.

To further verify the PCFC relevance of the cathodic polarization behavior, additional symmetric cell EIS measurements were conducted using proton-conducting BZCYYb electrolyte with the configuration PBSCFM_*x*_|BZCYYb|PBSCFM_*x*_. As shown in Figure S9, the BZCYYb-based symmetric cells exhibit the same composition-dependent trend as the GDC-based symmetric cells. PBSCFM01 shows the lowest *R*_p_ among the investigated cathodes, while PBSCFM03 remains superior to that of pristine PBSCF but inferior to that of PBSCFM01. These results confirm that the Mo-induced enhancement is preserved under a proton-conducting electrolyte configuration and is therefore directly relevant to the PCFC cathode operation.

Adequate electrical conductivity is one of the essential prerequisites for a material to qualify as a cathode in fuel cell applications. In general, electrode materials should exhibit an electrical conductivity exceeding 1 S/cm to be considered practical.^42^ Figure 7b illustrates the variation in electrical conductivity of PBSCFM_*x*_ (*x* = 0, 0.01, and 0.03) with temperature in dry air, where σ is plotted against the reciprocal of temperature (1000/T, in K^–1^). The electrical conductivity of the materials initially increases with rising temperature, reaching a maximum at approximately 250 °C for PBSCFM01 and around 300 °C for PBSCFM03, indicating semiconductor-like behavior. Beyond approximately 250 and 300 °C, respectively, the conductivity gradually decreases, reflecting a metallic behavior. The conduction mechanism of PBSCFM_*x*_ samples is characterized by a hopping *p*-type small polaron model (Co^4+^/Fe^4+^-O^2–^-Co^3+^/Fe^3+^),^33,43^ whereby charge carriers (electrons or holes) migrate between localized states, such as the mixed-valence states of transition metals (e.g., Fe^3+^/Fe^4+^ and Co^3+^/Co^4+^). With increasing temperature, thermal energy lowers the activation barrier for hopping, thereby thermally activating the localized electronic carriers and enhancing conductivity. However, after reaching the maximum conductivity, further temperature increase promotes oxygen release from the lattice and the partial reduction of Co^4+^/Fe^4+^ to Co^3+^/Fe^3+^, which decreases the concentration of electron holes responsible for *p*-type conduction. In addition, the oxygen vacancies generated at elevated temperature may interrupt the continuous B–O–B hopping pathway and increase lattice disorder, thereby suppressing small-polaron transport. The incorporation of Mo enhanced the electrical conductivity, with the PBSCFM01 sample achieving the maximum value of 1051 S/cm at 250 °C. However, further increases in Mo concentration result in a decline in conductivity (981 S/cm at 300 °C for PBSCFM03). The lower conductivity of PBSCFM03 compared with PBSCFM01 can therefore be attributed to the stronger charge-compensation effect of higher Mo^6+^ substitution, which reduces the hole concentration and partially dilutes the electronically active Co/Fe transport network.

As shown in Figure 8a, the cell exhibits a well-defined multilayer configuration consisting of a 400 μm Ni-BZCYYb anode support substrate with sufficient porosity, a dense BZCYYb electrolyte, and a 25 μm porous PBSCFM01 cathode uniformly deposited on top. SEM image of cross-sectional Ni-BZCYYb |BZCYYb| PBSCFM03 single cell is shown in Figure S10. By employing the same preparation process, the electrolyte layers of all cells with other two cathodes maintain nearly identical thicknesses, suggesting that the variations in electrochemical behavior are predominantly governed by differences in the cathode materials. To investigate the effect of different Mo doping concentrations, the electrochemical performance of the cell was tested under 3 vol %H_2_O humidified hydrogen supplied to the anode and dry ambient air fed to the cathode. The corresponding current–voltage-power (I–V–P) curves of the cell with PBSCFM01 and PBSCFM03 are illustrated in Figure 8b,c, respectively. As shown in Figure 8b, the PPDs of the PBSCFM01 cell are 817, 725, 628, and 525 mW·cm^–2^ at the temperature of 650, 600, 550, and 500 °C, respectively. In comparison, the PBSCFM03 cell achieves lower values of 706, 606, 506, 438 mW·cm^–2^ at the same operating temperature. This suggests that excessive Mo doping cannot guarantee better performance. These findings indicate that excessive Mo-substitution exerts a detrimental influence on cathodic performance, implying an optimal doping level exists for maximizing electrochemical activity. From Figure 8d, the PPD of PBSCFM01 cell increased ∼44% compared to that of the undoped PBSCF cell (Figure S11). Furthermore, as shown in Figure S12a, the long-term durability test of PBSCFM01 cell was operated at 600 °C under constant current density of 500 mA cm^–2^ with 3 vol %H_2_O–H_2_ at the anode and dry air at the cathode. The cell voltage remained stable over 120 h, indicating good operational stability of PBSCFM01 under the tested conditions.

**Figure 8:**
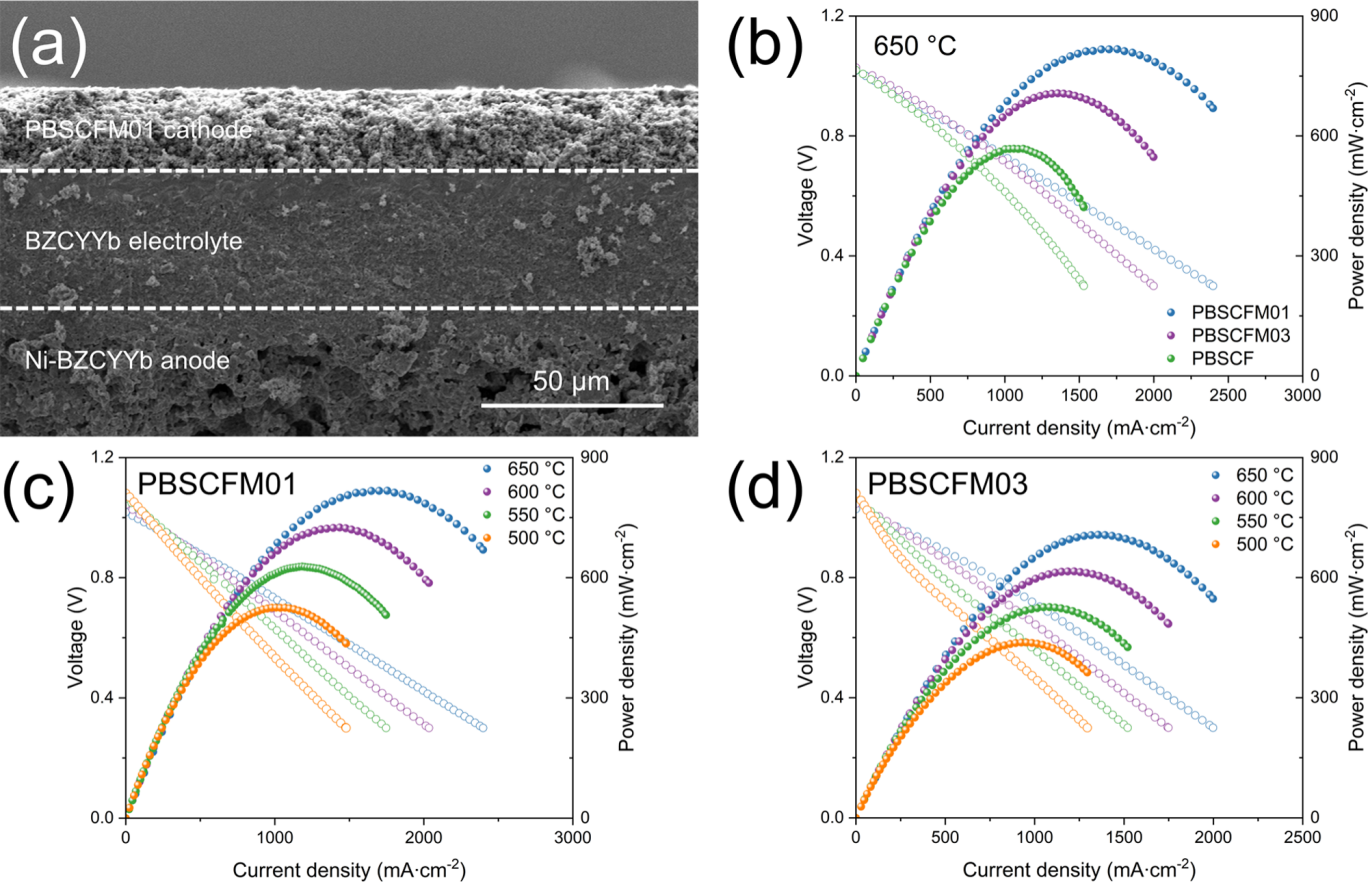
(a) SEM image of cross-sectional Ni-BZCYYb |BZCYYb| PBSCFM01 single cells; (b) PPDs comparison of single cells with the PBSCF, PBSCFM01, and PBSCFM03 cathode at 650 °C; (c) current–voltage–power (I–V–P) curve of the PBSCFM01 single cell at 500–650 °C; (d) I–V–P curve of the PBSCFM03 single cell at 500–650 °C.

## 4 Conclusions

In summary, this work has successfully prepared and evaluated high-performing cathode materials for PCFC—PBSCFM_*x*_ (*x* = 0, 0.01, and 0.03) by partially doping Mo into the B-site Fe cations of the perovskite oxide lattice. Results indicate that both PBSCFM01 and PBSCFM03 exhibit a pure-phase double perovskite structure. Both cathodes exhibit superior chemical compatibility with doped CeO_2_-based and BaZrO_3_-based electrolytes. XPS analysis shows that cobalt and iron ions coexist as Co^3+^/^4+^ and Fe^3+^/^4+^ in the PBSCFM_*x*_ samples. Aliovalent substitution of Mo^6+^ for Fe^3+^/^4+^ facilitates oxygen vacancies formation and enhances ORR kinetics. DFT results show that Mo doping reduces oxygen vacancy formation energy in PBSCFM01 and PBSCFM03. The peak electrical conductivity value of PBSCFM01 is 1051 S/cm at 250 °C. The *R*_p_ of PBSCFM_*x*_ cathodes are 0.242, 0.052, and 0.116 Ω·cm^2^ for PBSCF, PBSCFM01, and PBSCFM03 cathodes, respectively, at 650 °C in symmetrical cells with the GDC electrolyte. The PBSCFM01-based cell achieves a PPD of 817 mW·cm^–2^ at 650 °C, substantially outperforming the reference PBSCF cell, highlighting the positive effect of moderate Mo incorporation on electrochemical activity. Moreover, the PBSCFM01 cell exhibited excellent long-term durability, sustaining consistent performance over 120 h. In addition, PBSCFM01 exhibits the best electrical conductivity. Notably, characterizations reveal that excessive doping of Mo leads to a decline in the cathode performance. Hence, the optimal doping level should be designed meticulously to maximize cathode performance. These results demonstrate that PBSCFM01 possesses excellent potential as an efficient cathode material for PCFC applications, and that high-valence Mo doping represents an effective strategy for enhancing cathode performance.

## Supplementary Material



## Notes

XRD spectra of BZCYYb synthesized by the solid-state reaction method; Rietveld refinement and quantitative phase fraction of PBSCFM05; high-resolution TEM image of pristine PBSCF; microstructure characterization of the PBSCFM03 cathode; XPS spectra of Pr 3d for PBSCFM_x_; XPS spectra of Mo 3d for PBSCFM01 and PBSCFM03; oxygen nonstoichiometry of PBSCFM_x_ determined using TGA; SEM image of the cross-sectional PBSCFM03 |GDC| PBSCFM03 symmetrical cell; EIS results of PBSCFMx (*x* = 0, 0.01, and 0.03) cathodes; cross-sectional SEM image of the Ni-BZCYYb |BZCYYb| PBSCFM03 single cell; current–voltage–power (I–V–P) curve of the PBSCF single cell at 500–650 °C; and long-term stability test of a single cell with the PBSCFM01 cathode (PDF)
