# Supplementary material for: Mo Dopant-Mediated Oxygen Vacancy Engineering for Enhanced Cathodic Activity in Protonic Ceramic Fuel Cells
Source: ACS Appl Mater Interfaces. 2026 Jul 7;18(28):38703–15. doi: 10.1021/acsami.6c06472 (PMC13397490; doi:10.1021/acsami.6c06472)
Supplement: Supplementary file 1 [file am6c06472_si_001.pdf]

Supporting Information

Mo Dopant-Mediated Oxygen Vacancy Engineering for Enhanced Cathodic Activity in Protonic Ceramic Fuel Cells

Wenkai Yang[1, 3], Yuting Li[2, 3], Timileyin Aworinde[1, 3], Shaikh Abdullah[1], Lakshya Mathur[1, 3], Daofan Wang[1, 3], Yue Bao[4], Linfeng Yu[4], Lourdes Vega[2, 3], Chuancheng Duan[4], Sivaprakash Sengodan[1, 3, *]

[1]. *Department of Mechanical and Nuclear Engineering, Khalifa University, Abu Dhabi, UAE*

[2]. *Department of Chemical and Petroleum Engineering, Khalifa University, Abu Dhabi, UAE*

[3]. *Research and Innovation Center on CO_2 and Hydrogen (RICH), Khalifa University, Abu Dhabi, UAE*

[4]. *Department of Chemical Engineering, University of Utah, Salt Lake City, Utah 84112, United States*

[*] *Corresponding Author's email: sivaprakash.sengodan@ku.ac.ae*

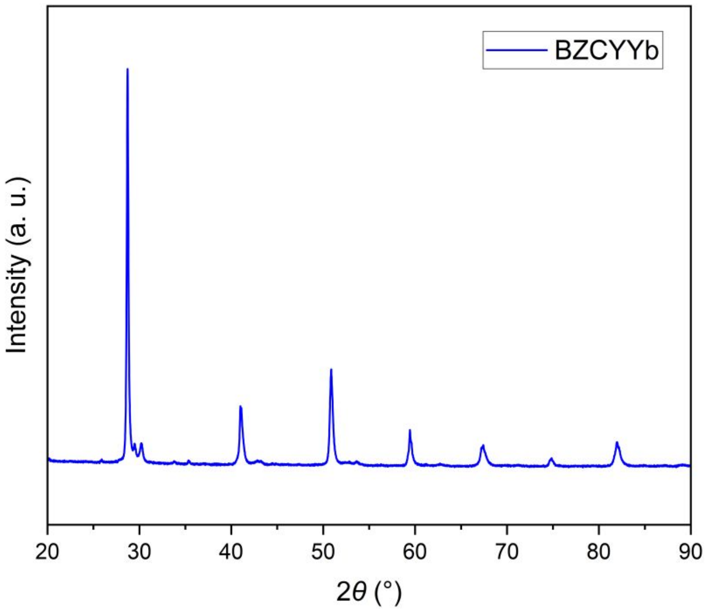

Figure S1. XRD spectra of BZCYYb synthesized by solid-state reaction method

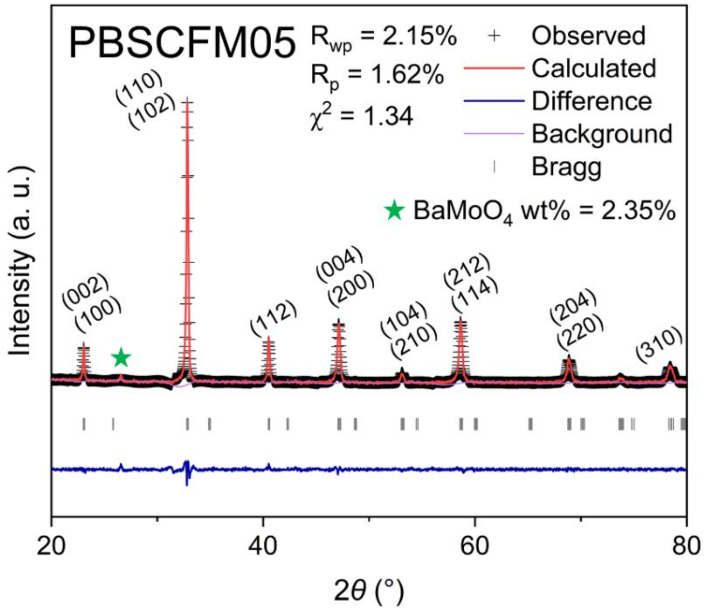

Figure S2. Rietveld refinement and quantitative phase fraction of PBSCFM05

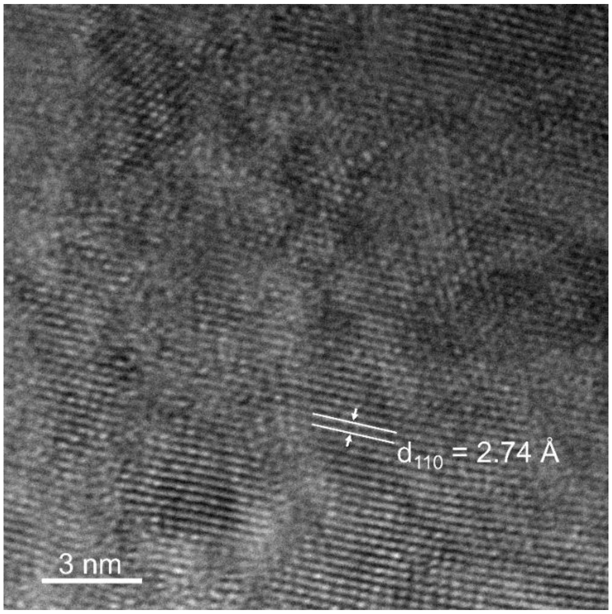

Figure S3. High-resolution TEM image of pristine PBSCF

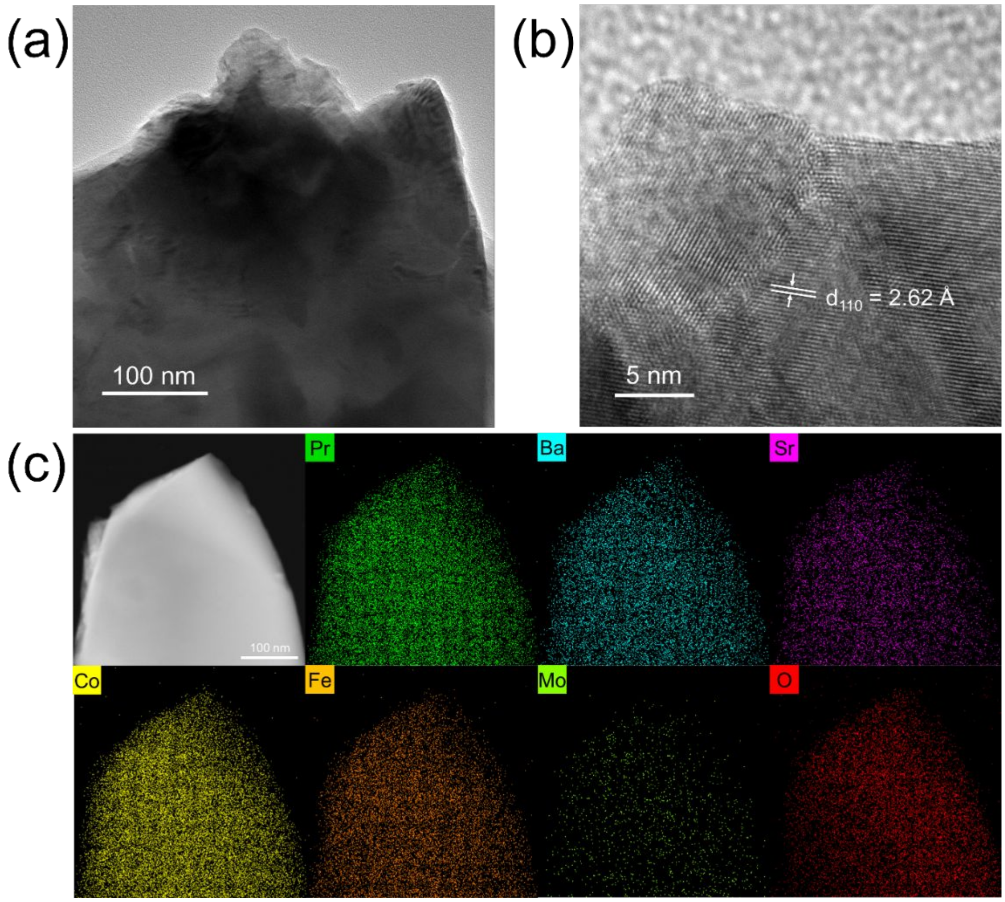

Figure S4. Microstructure characterization of PBSCFM03 cathode. (a) TEM image of PBSCFM03 powder; (b) High-resolution TEM image of PBSCFM03 powder in (a); (c) EDX-mapping results of PBSCFM03.

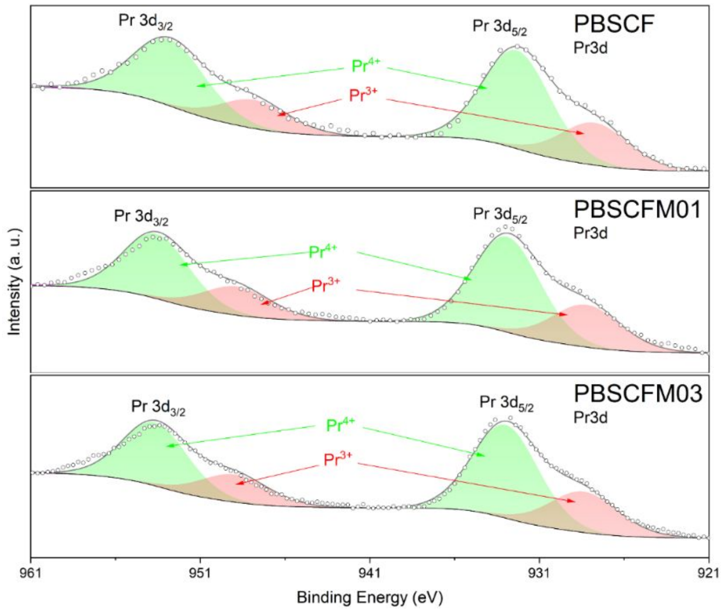

Figure S5. XPS spectra of Pr 3d for $PBSCFM_x$ (x = 0, 0.01, and 0.03)

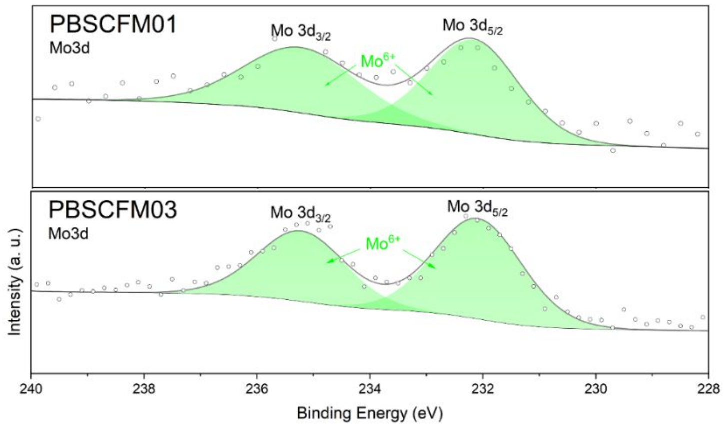

Figure S6. XPS spectra of Mo 3d for PBSCFM01 and PBSCFM03

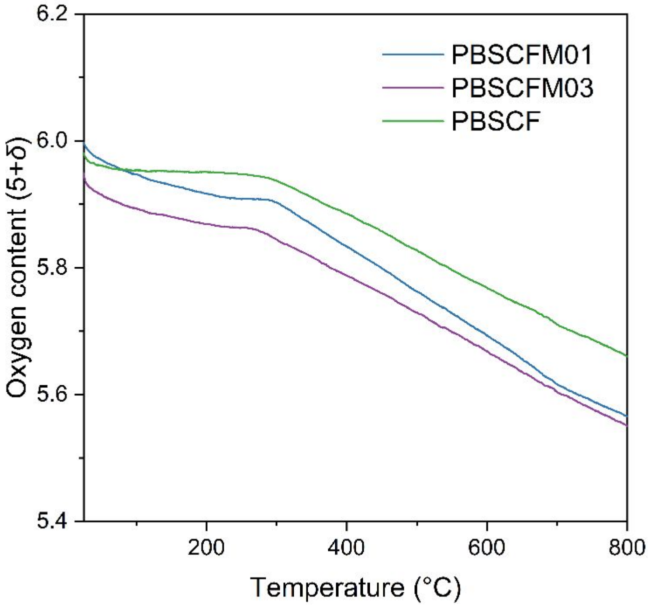

Figure S7. The oxygen nonstoichiometry of $PBSCFM_x$ determined using TGA. The initial oxygen contents at room temperature are determined by iodometric titration.

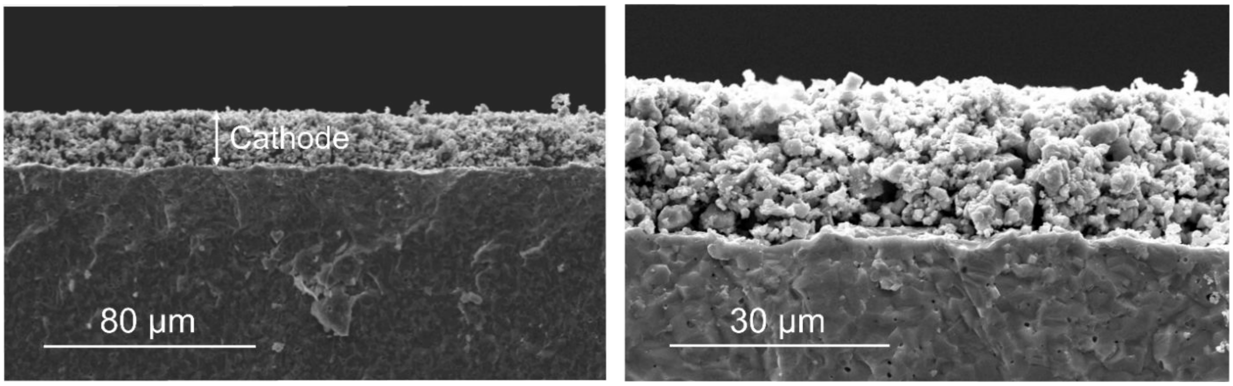

Figure S8. SEM image of cross-sectional PBSCFM03 |GDC| PBSCFM03 symmetrical cell

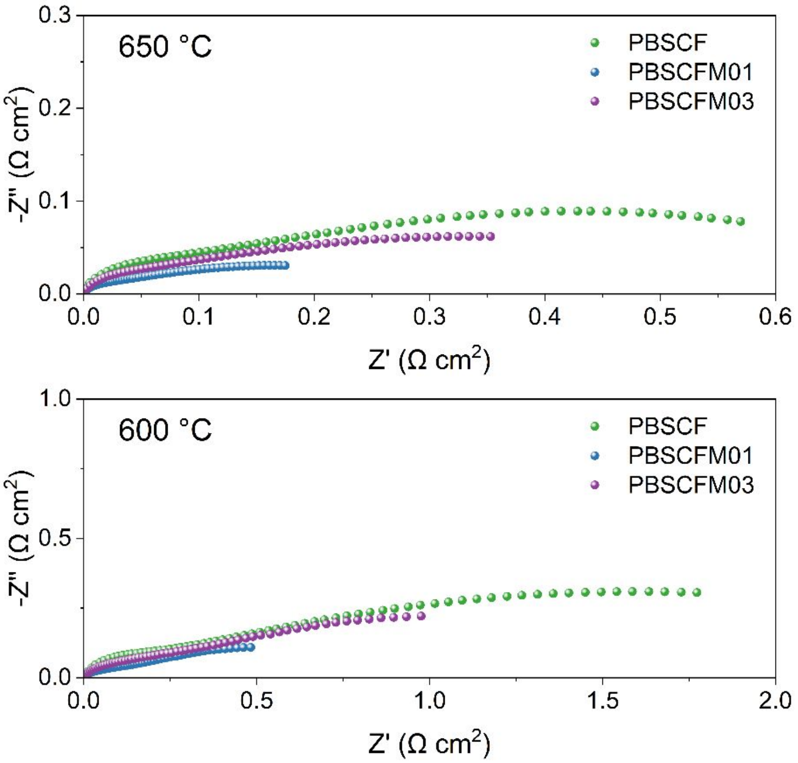

Figure S9. EIS results of $PBSCFM_x$ (x = 0, 0.01, and 0.03) cathodes on BZCYYb-based symmetric cells at 650 °C and 600 °C

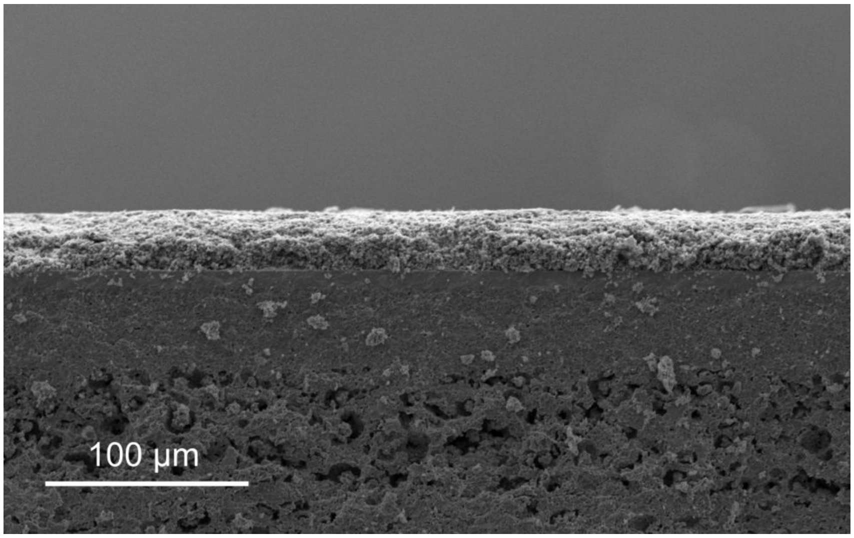

Figure S10. Cross-sectional SEM image of Ni-BZCYYb |BZCYYb| PBSCFM03 single cell

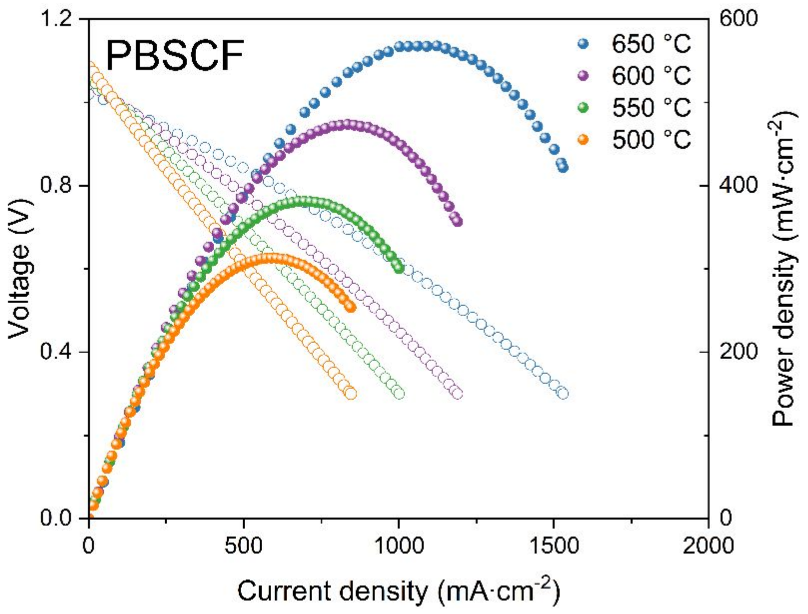

Figure S11. Current-Voltage-Power (I-V-P) curve of the PBSCF single cell at 500-650 °C

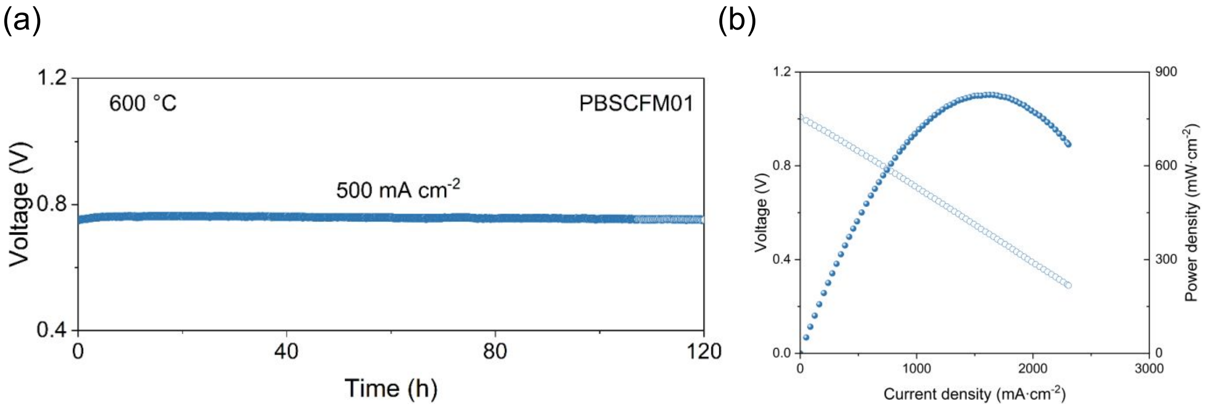

Figure S12. (a) Long-term stability test of a single cell with PBSCFM01 cathode at 600 °C; (b) Current-Voltage-Power (I-V-P) curve of the PBSCFM01 single cell at 650 °C before long-term stability testing

Table S1. The average oxidation states of B-site Co and Fe cations

Samples	Proportion of Fe Valence (%)		Average Oxidation States of Fe	Proportion of Co Valence (%)		Average Oxidation States of Co	Average Oxidation States of B-site
	Fe^{3+}	Fe^{4+}		Co^{3+}	Co^{4+}		
PBSCF	57.54	42.46	3.4246	43.11	56.89	3.5689	3.53
PBSCFM01	50.83	49.17	3.4917	57.78	42.22	3.4222	3.45
PBSCFM03	48.15	51.85	3.5185	55.09	44.91	3.4491	3.50

Table S2. The proportion of each species of Sr 3d

Sample	Proportion of Sr 3d (%)	
	Surface Sr	Lattice Sr
PBSCF	47.17	52.83
PBSCFM01	55.64	44.35
PBSCFM03	54.40	45.59

The Sr 3d spectra were reanalyzed using two constrained spin-orbit doublets corresponding to lattice Sr and surface-related Sr species. The lower-binding-energy doublet is assigned to Sr incorporated in the perovskite lattice, whereas the higher-binding-energy doublet is associated with surface Sr species, such as SrO_x/$SrCO_3$-like environments. The revised fitting results indicate that Mo incorporation modifies the surface Sr environment of PBSCF. However, because ex situ Sr 3d XPS provides surface chemical information rather than direct durability evidence, this result is suggestive of altered Sr surface chemistry rather than definitive proof of long-term stability.

Table S3 Iodometric titration data and absolute oxygen content at room temperature

**Sample**	**Trial 1 (mL)**	**Trial 2 (mL)**	**Trial 3 (mL)**	**Mean V (mL)**	**n($Na_2S_2O_3$) (mmol)**	**n(sample) (mmol)**	**Ratio**	**Baseline**	**δ**	**Abs. O content (5+δ)**	**SD(V) (mL)**	**SD(δ)**
**PBSCF**	6.70	6.40	6.70	6.60	0.660	0.2224	2.968	1.000	**0.984**	**5.984**	0.173	0.039
**PBSCF M01**	6.50	6.60	6.50	6.53	0.653	0.2222	2.941	0.960	**0.990**	**5.990**	0.058	0.013
**PBSCF M03**	6.30	6.30	6.10	6.23	0.623	0.2218	2.811	0.880	**0.965**	**5.965**	0.115	0.026

For iodometric titration, 0.1 g of sample was used for each measurement. A 0.1 M $Na_2S_2O_3$ standard solution was used as the titrant. The sample was treated with 15.0 mL of 4.0 M HCl and 10.0 mL of 20.0 wt% KI solution to provide an acidic environment and to liberate iodine through the oxidation of iodide ions. The released iodine was subsequently titrated with $Na_2S_2O_3$ solution, and the oxygen content was calculated from the consumed titrant volume.

Table S4. EIS results of three cathodes in the temperature range of 500-650 °C

R_p (Ω cm^2)	Temperature (°C)			
	650	600	550	500
PBSCF	0.242	0.394	0.723	1.494
PBSCFM01	0.052	0.081	0.146	0.317
PBSCFM03	0.116	0.205	0.385	0.713

Table S5. Comparison of peak power densities (PPDs) for Mo-doped cathode in single cell with other advanced fuel cell cathodes at 650 ℃ and 600 ℃

Cathode	Electrolyte	Anode	Peak power density ($mW·cm^{-2}$)		Ref.
			600 °C	650 °C	
PBSCFM01	BZCYYb	Ni-BZCYYb	725	817	This work
PBSCFM03	BZCYYb	Ni-BZCYYb	606	706	This work
$LSFNM_{0.05}$	BZCYYb	Ni-BZCYYb	172	300	[1]
SFM0.07	YSZ	Ni-YSZ	240	400	[2]
PSCFCM	YSZ	Ni-YSZ	/	~1000	[3]
SFMS	BCZY	Ni-BCZY	565	950	[4]
SFO-Mo	BCZY	Ni-BCZY	~400	~650	[5]
SFO-ZSSM	BCZY	Ni-CZY	854	1137	[5]
PBFM	BCZY	Ni-BCZY	~300	~500	[6]
PBFMN	BCZY	Ni-BCZY	795	897	[6]
SFM	YSZ	Ni-YSZ	/	260	[7]
SFMI	YSZ	Ni-YSZ	/	290	[7]
BSCFMo	BCZY	Ni-BCZY	~400	~760	[8]

$LSFNM_{0.05}$: $La_{0.6}Sr_{0.4}Fe_{0.85}Ni_{0.1}Mo_{0.05}O_{3-\delta}$; SFM0.07: $SrFe_{0.93}Mo_{0.07}O_{3-\delta}$; PSCFCM: $Pr_{0.4}Sr_{0.6}Co_{0.5}Fe_{0.4}Mo_{0.05}Cu_{0.05}O_{3-\delta}$; SFMS: $Sr_2Fe_{1.5}Mo_{0.25}Sc_{0.25}O_{6-\delta}$; BCZY: $BaCe_{0.7}Zr_{0.1}Y_{0.2}O_{3-\delta}$; SFO-Mo: $Sr_2Fe_{1.5}Mo_{0.5}O_6$; SFO-ZSSM: $Sr_2Fe_{1.5}Mo_{0.125}Sn_{0.125}Sc_{0.125}Zr_{0.125}O_6$; PBFM: $PrBaFe_{1.9}Mo_{0.1}O_{6-\delta}$; PBFMN: $PrBaFe_{1.7}Mo_{0.1}Ni_{0.2}O_{6-\delta}$; SFM: $Sr_2Fe_{1.5}Mo_{0.5}O_{6-\delta}$; SFMI: $Sr_2Fe_{1.5}Mo_{0.4}In_{0.1}O_{6-\delta}$; BSCFMO: $Ba_{0.5}Sr_{0.5}Co_{0.7}Fe_{0.2}Mo_{0.1}O_{3-\delta}$

To place the present cathode performance in the context of recently reported Mo-doped cathodes, Table S4 summarizes representative literature values for peak power density. The PBSCFM01

cathode delivers a peak power density of 817 mW·cm^{-2} at 650 °C, which are competitive with recently reported Mo-doped perovskite cathodes. Although direct comparison must consider differences in electrolyte thickness, cell configuration, active area, cathode loading, gas humidity, and testing conditions, the present PBSCFM01 cathode exhibits a favorable combination of low polarization resistance, high electrical conductivity, and high single-cell output. Compared with undoped PBSCF, the enhanced performance originates from the optimized low-level Mo substitution, which promotes oxygen-vacancy-related oxygen activation while maintaining phase purity and sufficient electronic conductivity. The lower performance of PBSCFM03 further highlights that the beneficial effect of Mo is non-monotonic and that excessive Mo incorporation can reduce the advantage by decreasing electronic carrier concentration and weakening the optimal defect balance.

Reference

(1) Ting, T.; Xie, D.; Chen, L.; Wang, L.-j. Effect of B-site Mo doping on electrochemical properties of La0.6Sr0.4Fe0.9Ni0.1O3−δ cathode materials for proton-conducting solid oxide fuel cells. *Transactions of Nonferrous Metals Society of China* **2025**, *35* (8), 2711–2725. DOI: https://doi.org/10.1016/S1003-6326(25)66842-7.

(2) Li, Y.; Mushtaq, N.; Chen, Y.; Ye, W.; Zhuang, Z.; Singh, M.; Jing, Y.; Fan, L. Revisiting Mo-Doped SrFeO3-δ Perovskite: The Origination of Cathodic Activity and Longevity for Intermediate-Temperature Solid Oxide Fuel Cells. *Advanced Functional Materials* **2025**, *35* (3), 2411025. DOI: https://doi.org/10.1002/adfm.202411025.

(3) Yu, X.; Wang, Z.; Ren, R.; Qiao, J.; Sun, W.; Xu, C.; Sun, K. Heterovalent Cu2+/Mo6+ bimetallic doping strategy for enhanced ORR kinetics and Cr tolerance in solid oxide fuel cell cathodes. *Chemical Engineering Journal* **2025**, *524*, 168891. DOI: https://doi.org/10.1016/j.cej.2025.168891.

(4) Yang, S.; Gu, Y.; Yu, S. The effect of Mo and Sc dopants on the performance of an Sr2Fe2O6 cathode for use in proton-conducting solid oxide fuel cells. *Materials Science and Engineering: B* **2025**, *311*, 117836. DOI: https://doi.org/10.1016/j.mseb.2024.117836.

(5) Zhou, L.; Yin, Y.; Nematov, D.; Dai, H.; Gu, Y.; Yu, S.; Bi, L. A high-performance cobalt-free cathode for proton-conducting solid oxide fuel cells via multi-element doping in Sr2Fe2O6. *Sustainable Materials and Technologies* **2026**, *48*, e01936. DOI: https://doi.org/10.1016/j.susmat.2026.e01936.

(6) Yao, P.; Zhang, J.; Qiu, Q.; Zhao, Y.; Yu, F.; Li, Y. A dual modification strategy of highly active catalytic cathode for proton-conducting solid oxide fuel cell with Ni-doped PrBaFe1.9Mo0.1O6-δ. *Journal of Power Sources* **2024**, *606*, 234591. DOI: https://doi.org/10.1016/j.jpowsour.2024.234591.

(7) Ma, Y.; Zhang, L.; Zhu, K.; Zhang, B.; Peng, R.; Xia, C.; Huang, L. In3+-doped Sr2Fe1.5Mo0.5O6−δ cathode with improved performance for an intermediate-temperature solid oxide fuel cell. *Nano Research* **2024**, *17* (1), 407–415. DOI: 10.1007/s12274-023-6338-y.

(8) Li, X.; Liu, Y.; Liu, W.; Wang, C.; Xu, X.; Dai, H.; Wang, X.; Bi, L. Mo-doping allows high performance for a perovskite cathode applied in proton-conducting solid oxide fuel cells. *Sustainable Energy & Fuels* **2021**, *5* (17), 4261–4267, 10.1039/D1SE00876E. DOI: 10.1039/D1SE00876E.
